# Supplementary material for: Awareness of alcohol marketing, ownership of alcohol branded merchandise, and the association with alcohol consumption, higher-risk drinking, and drinking susceptibility in adolescents and young adults: a cross-sectional survey in the UK
Source: BMJ Open. 2019 Mar 14;9(3):e025297. doi: 10.1136/bmjopen-2018-025297 (PMC6530316; doi:10.1136/bmjopen-2018-025297)
Supplement: Author's manuscript [file bmjopen-2018-025297.draft_revisions.pdf]

BMJ Open is committed to open peer review. As part of this commitment we make the peer review history of every article we publish publicly available.

When an article is published we post the peer reviewers' comments and the authors' responses online. We also post the versions of the paper that were used during peer review. These are the versions that the peer review comments apply to.

The versions of the paper that follow are the versions that were submitted during the peer review process. They are not the versions of record or the final published versions. They should not be cited or distributed as the published version of this manuscript.

BMJ Open is an open access journal and the full, final, typeset and author-corrected version of record of the manuscript is available on our site with no access controls, subscription charges or pay-per-view fees (http://bmjopen.bmj.com).

If you have any questions on BMJ Open's open peer review process please email info.bmjopen@bmj.com

BMJ Open

Awareness of alcohol marketing, ownership of alcohol branded merchandise, and the association with alcohol consumption, higher-risk drinking, and drinking susceptibility in adolescents and young adults: A cross-sectional survey in the United Kingdom.

Journal:	*BMJ Open*
Manuscript ID	bmjopen-2018-025297
Article Type:	Research
Date Submitted by the Author:	08-Jul-2018

Complete List of Authors:	Critchlow, Nathan; University of Stirling, Institute for Social Marketing<br>Mackintosh, Anne Marie; University of Stirling Institute for Social Marketing<br>Thomas, Christopher; Cancer Research UK, Cancer Policy Research Centre<br>Hooper, Lucie; Cancer Research UK, Cancer Policy Research Centre<br>Vohra, Jyotsna; Cancer Research UK, Cancer Policy Research Centre

Keywords:	PUBLIC HEALTH, Health policy < HEALTH SERVICES ADMINISTRATION & MANAGEMENT, Child protection < PAEDIATRICS, SOCIAL MEDICINE

SCHOLARONE™
Manuscripts

For peer review only

Running head: Alcohol marketing and consumption in young people in the UK

Title: Awareness of alcohol marketing, ownership of alcohol branded merchandise, and the association with alcohol consumption, higher-risk drinking, and drinking susceptibility in adolescents and young adults: A cross-sectional survey in the United Kingdom.

Running head: Alcohol marketing and consumption in young people in the UK

Authors

Nathan Critchlow[1]

Anne Marie MacKintosh[1]

Christopher Thomas[2]

Lucie Hooper[2]

Jyotsna Vohra[2]

[1] Institute for Social Marketing, Faculty of Health Sciences and Sport, University of Stirling, Stirling.

[2] Cancer Policy Research Centre, Cancer Research UK, London.

Corresponding author

Nathan Critchlow, Institute for Social Marketing, Faculty of Health Sciences and Sport, University of Stirling, Stirling, FK9 4LA. E-mail: nathan.critchlow@stir.ac.uk; Tel: +44(0)1786467382.

Word Count: 3,910

Table and figure count: 5 (All tables)

For peer review only

For peer review only - http://bmjopen.bmj.com/site/about/guidelines.xhtml

Running head: Alcohol marketing and consumption in young people in the UK

Awareness of alcohol marketing, ownership of alcohol branded merchandise, and the association with alcohol consumption, higher-risk drinking, and drinking susceptibility in adolescents and young adults: A cross-sectional survey in the United Kingdom.

ABSTRACT

Objectives: To explore awareness of alcohol marketing in adolescents and young adults in the United Kingdom (UK), what factors are associated with awareness, and what association awareness has with alcohol consumption, higher-risk drinking, and drinking susceptibility?

Design: Online cross-sectional survey conducted April–May 2017.

Setting: UK.

Participants: Adolescents and young adults aged 11-19 years old in the UK (*n*=3,399) (average age: 15.18).

Main outcome measures: Score on Alcohol Use Disorders Identification Test–Consumption (AUDIT-C) (0-12) and indication of higher-risk consumption ($\geq$5 AUDIT-C) in current drinkers. Susceptibility to drink alcohol in the next year (Yes/No) in never drinkers.

Results: Eighty-two percent of respondents were aware of at least one form of alcohol marketing in the past month and 17% owned alcohol branded merchandise. Chi-square tests found that higher awareness of alcohol marketing was associated was being a current drinker (p<0.001), a higher-risk drinker (p<0.001), frequency of parental consumption (p<0.001), and perceived parental (p<0.001) and peer approval of consumption (p<0.001). Among current drinkers, hierarchical regressions (controlling for demographics and interpersonal correlates of consumption) found that marketing awareness and owning branded merchandise was positively associated with AUDIT-C score and higher-risk consumption. For example, current drinkers reporting medium marketing awareness were twice as likely to be higher-risk drinkers as those reporting low awareness (AOR=2.18, 95% CI: 1.39-3.42, p<0.001). Among never drinkers, respondents who owned branded merchandise were twice as likely to be susceptible to drinking as those who did not (AOR=1.98, 95% CI: 1.20-3.24, p=0.007).

Conclusions: Young people, above and below the legal purchasing age for alcohol in the UK, are aware of alcohol marketing through a range of channels and almost one-in-five own branded merchandise. Marketing awareness in current drinkers was independently associated with increased alcohol consumption and greater likelihood of higher-risk consumption. In never drinkers, ownership of branded merchandise was associated with greater susceptibility.

For peer review only - http://bmjopen.bmj.com/site/about/guidelines.xhtml

Running head: Alcohol marketing and consumption in young people in the UK

Keywords: Alcohol marketing; Young people; Higher-risk drinking; Alcohol Advertising; Susceptibility; Survey; Public Health; Health Policy.

Strengths and limitations of this study

- This is the first study in the UK to examine alcohol marketing awareness and ownership of alcohol branded merchandise in a demographically representative sample of young people across the UK (England, Scotland, Wales, and Northern Ireland), including those above and below the legal purchasing age for alcohol.
- The study provides timely insight into what forms of alcohol marketing young people are aware of in the current media landscape, how frequently they recall seeing alcohol marketing through these channels, and what factors are associated with higher awareness of alcohol marketing.
- The large sample size supports robust statistical analysis to examine what relationship (if any) there is between alcohol marketing awareness and consumption, controlling for other demographic and interpersonal correlates of alcohol consumption.
- The study explores the association between alcohol marketing and consumption at three levels: overall alcohol consumption, higher-risk consumption, and susceptibility in never-drinkers.
- The cross-sectional nature of the survey does not enable causal relationships to be drawn about the link between alcohol marketing and either alcohol consumption or susceptibility to drink.

For peer review only - http://bmjopen.bmj.com/site/about/guidelines.xhtml

Running head: Alcohol marketing and consumption in young people in the UK

Awareness of alcohol marketing, ownership of alcohol branded merchandise, and the association with alcohol consumption, higher-risk drinking, and drinking susceptibility in adolescents and young adults: A cross-sectional survey in the United Kingdom.

INTRODUCTION

Adolescents and young adults (young people) are a focal population for alcohol research because consumption at this stage of development is associated with increased consumption and risk of concomitant harms in later adulthood [1,2]. Global estimates indicate that consumption by young people is particularly high in Europe, where the proportion of current drinkers (69.5%) is higher than the five other global regions, and the proportion of lifetime abstainers is lower (15.9%) [3]. In England, it is estimated that approximately half of 11-15-year olds (44%) have consumed an alcoholic drink, one-in-ten have consumed in the past week, and nine percent have been drunk in the past month [4]. Similar estimates are reported in Scotland and Wales [5,6]. Understanding the drivers of alcohol consumption in young people is important given the immediate and long-term individual, social, and economic consequences associated with higher-risk drinking [7].

One factor routinely cited as shaping alcohol-related attitudes and behaviours in young people is marketing [8,9]. Marketing is fundamentally important to alcohol producers. It represents the primary method of communicating with new and existing consumers, can directly encourage sales, and can increase brand salience over competitors. Accordingly, alcohol companies have used highly visible marketing for over 100 years [10], with the current UK landscape characterised by a complex network of mass media marketing (e.g. television), alternative marketing (e.g. sponsorship), consumer marketing (e.g. price), and stakeholder marketing (e.g. retailers) [9]. The importance of marketing to the alcohol industry is evidenced through their annual investment, with Diageo's global marketing expenditure approximately £1.8 billion [11]. Continued consolidation in the alcohol industry has also seen

For peer review only - http://bmjopen.bmj.com/site/about/guidelines.xhtml

the market become dominated by a small number of transnational producers, creating larger marketing budgets, economies of scale, and intense competition [12].

Content analysis research, which focuses on marketing output as the unit of analysis, consistently reports that marketing may reach and influence young people. For example, marketing has been reported in media environments where young people may be exposed, including sports [13], social media [14], print media [15], and on-screen [16, 17]. Content research has also found that marketing may appeal to young people through creative designs, topical and real-world associations, and positive connotations around consumption such as sociability or desirable lifestyles [18,19]. It has also been suggested that commercial marketing contains ambiguous messages about lower-risk consumption [20,21].

Systematic reviews of consumer research, which focus on the individual as the unit of analysis, provide consistent evidence that awareness of, and participation with, marketing has a causal influence on consumption, including initiation and frequency of drinking [22,23]. Message interpretation research has also attempted to move the debate on from whether marketing is associated with consumption and onto how this influence occurs, by identifying psychological mechanisms which mediate the relationship between exposure and consumption [24,25].

In the UK, the influence of alcohol marketing on young people, and the appropriate regulatory response, has been a topic of debate for decades [9,26]. There are, however, unresolved issues which have inhibited attempts to move the debate forward. The last large-scale assessment of young people's awareness of alcohol marketing in the UK is a decade old, was only conducted in Scotland, only sampled adolescents under the minimum purchase age, only considered overall marketing awareness (not frequency), and did not consider higher-risk consumption [27,28]. In this study, we explore frequency of awareness for alcohol marketing and ownership of alcohol branded merchandise in young people above and

For peer review only - http://bmjopen.bmj.com/site/about/guidelines.xhtml

below the legal purchasing age, and consider what association (if any) this has with alcohol consumption, likelihood of higher-risk drinking, and susceptibility to drink in the next year, in a demographically representative sample in the UK.

METHOD

Design and sample

Data come from the 2017 Youth Alcohol Policy Survey, an online cross-sectional survey conducted with 11-19-year olds in the UK (*n*=3,399). Responses were collected April–May 2017. The survey was hosted by YouGov, a market research company, who recruited a representative sample from their UK panel [29]. The study design was informed by previous cross-sectional surveys in the UK exploring young people's experiences of alcohol and tobacco marketing [27,30].

Measures

Demography

Age, gender, ethnicity, resident country (England, Scotland, Wales, Northern Ireland), living status, employment status, educational status, legal purchasing status, and indices of deprivation (IMD), were obtained from information held about panel respondents or survey questions.

Awareness of alcohol marketing

Awareness of alcohol marketing was assessed through structured, self-reported recall, a method frequently used in consumer research [31]. Participants were prompted with the statement '*Over the last month, how often, if at all, have you seen…*' and then presented with descriptions of marketing in nine channels: (1) newspapers or magazines; (2) television; (3)

For peer review only

billboards; (4) radio; (5) adverts on social media (e.g. YouTube, Tumblr, Facebook, SnapChat, Instagram or other social media); (6) famous people in films, music videos or TV or pictured in magazines; (7) sports, games, or events sponsorship; (8) special price offers; and (9) competitions or prize draws. For each channel, a six-point Likert scale was used to measure frequency of noticing marketing in the past month (1=Everyday – 6=Not in the past month; Not sure).

For each channel, the self-reported frequency of awareness was converted into the estimated number of days that marketing had been seen in a four-week period (i.e. 'one month'). For example, an answer of 'everyday' for television advertising equated to 28 instances of awareness over four weeks (i.e. seven days a week multiplied by four). Scores across the nine channels were summed to create an aggregate score, providing an approximation of the number of times that participants had noticed alcohol marketing in the past month. This aggregate score could only be computed for cases where a valid answer had been given for all nine channels. If a participant answered 'not sure' to any of the nine channels they were coded as 'not stated' for the aggregate score. The aggregate score for the valid sample was split into tertiles of low (aggregate score ≤16), medium (17-53) and high awareness (≥54).

Ownership of alcohol brand merchandise

Participants were asked '*Do you own any merchandise (such as clothing or drinks glasses) that show an alcoholic drink brand or logo?*' (Yes/No/Not sure).

Drinking status

Participants were asked '*Have you ever had a whole alcoholic drink? Not just a sip.*' Those who answered 'No' were classed as never-drinkers while those who answered 'Yes' were

For peer review only - http://bmjopen.bmj.com/site/about/guidelines.xhtml

For peer review only

classed as ever-drinkers. Ever-drinkers were also asked '*How often do you usually have a drink containing alcohol*?' with response options of 'Never', 'Monthly or less often', '2-4 times a month', '2-3 times a week', and '4 or more times a week'. Those who answered other than 'Never' were classed as current-drinkers.

Alcohol consumption and higher-risk drinking

Alcohol use was measured through the Alcohol Use Disorders Identification Test–Consumption (AUDIT-C), which assessed frequency of consumption, units drunk in a typical occasion, and frequency of high episodic drinking. Responses were provided on five-point scales with the answers for each item relative to frequency (0=Never – 4= Four or more times a week), units drunk (0=1-2 units – 4=10 units or more), and frequency of high episodic drinking (0=Never – 4=Daily or almost Daily). High episodic drinking was classified as eight or more units in a single occasion for males, and six or more units for females (one unit=8g or 10ml of alcohol). A diagram depicting the unit content of alcoholic drinks was included to assist calculation of units. A total AUDIT-C score was computed (0-12), with a cut-off of ≥5 used to identify higher-risk consumption [32].

Susceptibility

As per tobacco research, susceptibility was defined as the absence of a firm decision not to drink alcohol in the next year [30]. Never-drinkers were classified as 'non-susceptible' if they answered 'definitely no' to the question '*Do you think you will drink alcohol at any time during the next year*?' Those who answered anything other than 'Definitely no' were classified as 'Susceptible'.

Confounding variables

For peer review only - http://bmjopen.bmj.com/site/about/guidelines.xhtml

Running head: Alcohol marketing and consumption in young people in the UK

Confounding factors were included to contextualise the association (if any) between marketing and consumption. Frequency of consumption was measured for the mother (female carer), father (male carer), and closest friend (each scored: 1=Never – 9=Every day or almost every day; Prefer not to say; Not applicable). For each group, self-reported consumption was also converted into the estimated number of days alcohol was consumed on each year (e.g. every day or almost every day equated to 312 days per year). Perceived acceptability of consumption was measured for parents and peers (each scored: 1=Total acceptable – 5=Totally unacceptable). For both groups, acceptability was converted into dichotomous categories ('Neutral or unacceptable' and 'Acceptable'). For ever drinkers, age of first drink was also measured (≤8 years old – 19 years old; Can't remember; Prefer not to say). Answers were converted into three categories (≤13 years; 14-15 years; ≥16 years).

Ethics

Ethical approval was obtained from the University of Stirling's General University Ethics Panel (GUEP59). YouGov included a lead for ethical and quality assurance, including consent, post-survey debriefing and signposting to support organisations, and confidentiality and anonymity.

Analysis

Data were analysed using SPSS version 23. Descriptive data were weighted so percentages and median scores were representative of the demographic profile of young people in the UK. Bivariate analyses, using Chi-square tests, were conducted to examine the relationship between alcohol marketing awareness and demographic and confounding variables.

Multivariate analyses were conducted on unweighted data, as demographic and confounding variables were controlled for in the regressions. A hierarchical linear regression

For peer review only - http://bmjopen.bmj.com/site/about/guidelines.xhtml

was conducted with current drinkers' AUDIT-C score as the dependent variable and awareness of marketing and ownership of branded merchandise as key independent variables. The following demographic and confounding variables were also included in initial blocks: age; gender; ethnicity; IMD quintile; resident country; educational status; working status; living status; frequency of mother (female carer), father (male carer) and close friend drinking; perceived parental and peer acceptability of consumption; and drink age of first drink. Categorical variables with $\geq$3 categories were converted into dummy (binary) variables to aid interpretation and comparison. The omitted dummy variable formed the reference category. For example, marketing awareness was a categorical variable with four levels: low, medium, high, and not stated. Four binary variables were computed: low awareness, medium awareness, high awareness, and not stated (each coded Yes=1, No=0). By including medium, high and not stated in the regression analysis, and omitting low awareness, the reference category was low awareness. The regressions therefore indicate the association between level of consumption and medium awareness, relative to low awareness, and high awareness relative to low awareness. Reference categories for each variable are displayed in results (Table 3).

Two hierarchical logistic regressions were conducted with higher-risk drinking (AUDIT-C $\geq$5) among current drinkers and susceptibility to drink among never-drinkers as the dependent variables. Marketing awareness and ownership of branded merchandise were the key independent variables. Where applicable, both logistic regressions controlled for the same demographic and confounding variables as the linear regression. Reference categories for categorical independent variables are indicated in the results (Tables 4 and 5). Where the categorical variables had three $\geq$3 levels, and were of an ordinal level, the SPSS contrast=difference function enabled comparison of each increasing category relative to the combined previous categories. For example, the first comparison with frequency of mother's

For peer review only - http://bmjopen.bmj.com/site/about/guidelines.xhtml

drinking and higher-risk drinking was 'less than monthly drinking' vs. 'mother never drinks', whereas the final comparison was 'at least weekly drinking' vs. 'less often'. As the independent variables were categorical, 'not stated' responses were also included as a separate category and compared against the reference category for each variable. This enabled the maximum sample to be retained. For example, the large number of 'not stated' responses on level of marketing awareness could be compared with those for whom marketing awareness could be computed.

RESULTS

Sample characteristics

The weighted sample (*n*=3,399) had an average age of 15.18 years old (*SD*=2.55; range: 11-19), with three quarters (76%) below the legal purchasing age (≤18 years). There was an even distribution for gender (51% female and 49% male). The majority of the sample were White British (76%) and were evenly distributed across IMD (20% in each quintile). Most participants lived in England (84%) with the remainder from Scotland (8%), Wales (5%), and Northern Ireland (3%). Almost all participants were living at home with parent(s) or other adult family members (90%) and were in some form of education (95%).

Awareness of alcohol marketing

The most frequent sources of marketing awareness in the past month were adverts on television (Median 6 instances per month, Inter quartile range=14), celebrity endorsement (Median 6, IQR=14), and special offers (Median 6, IQR=14) (Table 1). More than a third of participants (%) had noticed marketing through these channels at least weekly. Billboard adverts (Median 2 instances per month, IQR=6) and social media adverts (Median 2, IQR = 6) were noticed less than once a week, with at least a quarter of participants (%) having

For peer review only - http://bmjopen.bmj.com/site/about/guidelines.xhtml

noticed such adverts at least weekly. Lowest awareness was for adverts in the print press (Median 0 instances per month, IQR=6), on radio (Median 0, IQR = 0) and competitions (Median 0, IQR=2). For each of the channels, a fifth or more (range: 19-29%) were not sure how often, if at all, they had come across alcohol marketing. Overall, 82% had noticed marketing through least one channel.

[TABLE 1]

Aggregate alcohol marketing awareness

The median aggregate alcohol marketing score was 32 (IQR=60), equating to noticing 32 instances of alcohol marketing in the past month (under minimum purchase age: median=28; IQR=60). When categorised into tertiles, 35% of the valid sample were classified as having low awareness (≤16 instances per month), 32% had medium awareness (17–53), and 34% had high awareness (≥54). In those under the minimum purchase age, 38% had low awareness, 31% medium, and 32% high. Bivariate Chi-square tests found that higher awareness was significantly associated with being male, of legal purchasing age, a current drinker, a higher-risk drinker, not in education, in employment, and perceiving parents and peers would consider it okay to consume (Table 2). High awareness was also associated with greater frequency of mother (female carer) consumption, $\chi^2(16)=38.25$, $p<0.001$, and greater frequency of father (male carer) consumption, $\chi^2(16)=198.51$, $p<0.001$. There was no difference in awareness by ethnicity, IMD quintile, or resident country.

[TABLE 2]

Owning alcohol branded merchandise

For peer review only - http://bmjopen.bmj.com/site/about/guidelines.xhtml

Running head: Alcohol marketing and consumption in young people in the UK

Almost a fifth of participants (17%) reported owning branded merchandise.

Association between alcohol marketing and AUDIT-C scoring

Almost half of the sample (48%; *n*=1,592) were current drinkers. Within current drinkers, the average AUDIT-C score was 4.30 (*SD*=2.76). A hierarchical linear regression, controlling for demographic and confounding variables, was conducted to examine the association between marketing awareness, ownership of branded merchandise, and AUDIT-C scoring.

In the final stage of the model, of the demographic variables, being older (*b*=0.43, 95% CI: 0.35-0.51, *p*<0.001), male (*b*=0.31, 95% CI: 0.09-0.54, *p*=0.006), from a more affluent IMD (*b*=0.11, 95% CI: 0.03-0.20, *p*=0.007), in education (*b*=0.66, 95% CI: 0.55-1.10, *p*=0.003), and living independently of parents or adult family members (*b*=0.87, 95% CI: 0.54-1.20, *p*<0.001) was associated with higher AUDIT-C score (Table 3). Of the confounding variables, having a close friend who drinks at least weekly (*b*=1.44, 95% CI: 1.19-1.69, *p*<0.001), and perceiving that parents consider it acceptable to consume (*b*=0.29, 95% CI: 0.01-0.57, *p*=0.39) was associated with higher AUDIT-C score. Having a first alcoholic drink at 16 years old or over (*b*= -1.33, 95% CI: -1.63 – -1.04, *p*<0.001) was associated with lower AUDIT-C score, compared with those who first drank aged 14–15 years. Of the marketing variables, medium awareness (*b*=0.79, 95% CI: 0.37-1.21, *p*<0.001), or high awareness (*b*=0.85, 95% CI: 0.44-1.26, *p*<0.001), compared to low awareness, was associated with higher AUDIT-C score, as was ownership of branded merchandise (*b*=0.79, 95% CI: 0.55-1.04, *p*<0.001).

[TABLE 3]

Association between alcohol marketing and higher-risk consumption

For peer review only - http://bmjopen.bmj.com/site/about/guidelines.xhtml

Running head: Alcohol marketing and consumption in young people in the UK

Almost half of current drinkers (44%; *n*=699) were classified as higher-risk (≥5 AUDIT-C). A hierarchical logistic regression, controlling for demographic and confounding variables, was conducted to examine the association between marketing awareness, ownership of branded merchandise, and higher-risk drinking.

In the final stage of the model, of the demographic variables, being older (Adjusted Odds Ratio=1.40, 95% CI: 1.28-1.53, p<0.001), male (AOR=1.32, 95% CI: 1.04-1.68, p=0.022), from England compared to Wales (AOR=0.58, 95% CI: 0.36-0.93, p=0.024), in education (AOR=1.61, 95% CI: 1.01-2.55, p=0.045), and living independently (AOR=1.56, 95% CI: 1.09-2.23, p=0.015) was associated with higher-risk drinking (Table 4). Of the confounding variables, increasing frequency of close friend consumption (p<0.001), and having had first drink aged 14-15 years old or younger (AOR=0.26, 95% CI: 0.19-0.35, p<0.001) was associated with higher-risk consumption. Of the marketing variables, medium awareness (AOR=2.18, 95% CI: 1.39-3.42, p<0.001), high awareness (AOR=1.43, 95% CI: 1.01-2.02, p=0.045), and owning branded merchandise were associated with higher-risk drinking (AOR=1.81, 95% CI: 1.31-2.22, p<0.001).

[TABLE 4]

Association between alcohol marketing and susceptibility to consume

Half of participants (52%) were classified as 'never drinkers'. Within never drinkers, half (52%; *n*=830) were susceptible to consumption (i.e. did not definitively reject that they would consume in the next year). A hierarchical logistic regression, controlling for demographic and confounding variables, was conducted to examine the association between marketing awareness, ownership of branded merchandise, and susceptibility.

For peer review only - http://bmjopen.bmj.com/site/about/guidelines.xhtml

Running head: Alcohol marketing and consumption in young people in the UK

In the final stage of the model, of the demographic variables, only being white British (AOR=1.51, 95% CI: 1.12-2.03, p=0.07) was associated with susceptibility (Table 5). Of the confounding variables, frequency of mother (female carer) consumption (p<0.001), frequency of father (male carer) consumption (p=0.023), frequency of close friend consumption (p<0.001), and perceived peer approval (AOR=2.29, 95% CI: 1.77-2.96, p<0.001) were associated with susceptibility. Of the marketing variables, awareness of alcohol marketing was not significantly associated with susceptibility, but ownership of branded merchandise was (AOR=1.98, 95% CI: 1.20-3.24, p=0007), with those who owned branded merchandise being more likely to be susceptible.

[TABLE 5]

DISCUSSION

The findings indicate that young people are aware of a variety of alcohol marketing, that awareness is associated with increased consumption and higher-risk drinking in current drinkers, and that ownership of branded merchandise is associated with susceptibility in never-drinkers. We address key evidence gaps in the UK by exploring frequency of marketing awareness over a one-month period and demonstrating an association between marketing and both consumption and susceptibility in a demographically representative sample of young people above and below the legal purchasing age.

The findings are consistent with suggestions that alcohol marketing appears in contexts which may reach young people, including those under the legal purchasing age [8,9]. That awareness included mass media marketing (e.g. television), alternative marketing (e.g. sponsorship and celebrity endorsement), consumer marketing (e.g. price offers), and new media marketing highlights the dynamic nature of '360-degree' marketing strategies and how

For peer review only - http://bmjopen.bmj.com/site/about/guidelines.xhtml

they reach young people in offline and online environments [9,33]. By measuring frequency of awareness, the results extend understanding by showing how often young people saw marketing; with at least one-in-ten reporting daily or almost daily awareness through three of the nine channels. That frequency of awareness was particularly high for television and celebrity endorsement is consistent with research which has found that alcohol appears frequently in popular television shows and sports broadcasts [13,17]. Approximately half of the sample had seen at least 32 instances of marketing per month, which equates to at least one a day. Although there were expected differences in awareness between drinkers and never-drinkers [27], there were no differences between key demographic groups, including ethnicity, indices of deprivation, and resident country. This suggests that exposure to marketing occurs in young people across the UK, and is not isolated to a minority of demographic groups.

The results are consistent with longitudinal research which has shown a link between marketing and increased consumption in young people [22,23]. Although marketing awareness did not have an association with susceptibility in never-drinkers, ownership of branded merchandise did. Research has reported that participation with marketing has a stronger association with consumption than awareness [27,28,34]. Our findings therefore suggest that the effect of participation is pronounced in never-drinkers. The findings also extend understanding by showing an association between marketing and consumption across young adulthood. This includes an association with susceptibility and consumption in young people under the legal purchasing age and higher-risk drinking in newly-legal drinkers. Newly legal drinkers are an important target for alcohol marketers [18] and are a key under-researched group [35]. The findings therefore highlight the importance of considering the wider role that marketing plays on consumption, not just under the purchasing age.

For peer review only - http://bmjopen.bmj.com/site/about/guidelines.xhtml

Except for the Scottish Government's decision to implement minimum unit pricing [36], there has been little change to the UK's self-and-co-regulatory framework for alcohol marketing [37,38]. It is claimed that such self-regulatory approaches provide inadequate restrictions, are not suitably enforced, are retrospective and slow to react to complaints, and lack meaningful sanctions [9,39-41]. Although statutory regulations are cited as an alternative approach [26], studies have also questioned whether current examples, such as the Loi Évin in France, are being enforced properly or whether they reduce marketing exposure [13,31]. Further research exploring the perspectives of stakeholders involved in the production, research, consumption, and regulation of marketing would be of value to identify feasible and effective options to reduce youth exposure and form a consensus on appropriate action [42,43].

There are limitations and directions for future research. The cross-sectional design cannot identify a causal relationship between marketing and consumption, albeit a directional effect is supported by longitudinal research [22,23]. That marketing had any association with consumption and susceptibility, after controlling for confounding variables, suggests that marketing must at least play either an initiating or reinforcing role. The study also only sampled teenagers (≤19 years old) and, consequently, the results are only partially representative of legal purchasing adults, albeit other research has shown similar trends in older young adult populations [34]. This study only explored a direct association between marketing and consumption. Qualitative research suggests that young people's relationship with marketing is more complex than an 'exposure equals consumption' hypothesis, and that marketing holds cultural, social, and symbolic meaning [44,45]. Future research, based on message interpretation process models [24], should explore the psychological and social cognitive pathways which mediate the association between marketing and consumption.

For peer review only - http://bmjopen.bmj.com/site/about/guidelines.xhtml

Compared to other studies, the marketing channels measured are not exhaustive and the results may underestimate awareness. Examples of omitted marketing include packaging, cinema, product placement, point of sale, and a broader range of digital marketing [27,34,46]. It was also not possible to decipher whether 'not sure' responses indicated uncertainty over whether a participant had seen alcohol marketed through that channel at all or whether they were unsure on their frequency of awareness. This influenced the design of the regression models (to account for a 'Not sure' category). Finally, except for owning branded merchandise, the study only measured awareness of marketing, but not participation. As participation is reported to have a stronger association with consumption [27,34,46], the results may underestimate the association between marketing and consumption. Future iterations of the Youth Alcohol Policy Survey should refine measurement tools to account for participation and mediating social and marketing specific cognitive factors.

CONCLUSION

This paper makes important contributions to understanding by exploring alcohol marketing awareness, ownership of branded merchandise, and the association with consumption in a representative sample of young people in the UK, three quarters of who were under the legal purchasing age. The results highlight that '360-degree' marketing strategies have created several avenues for young people to be exposed to, or involved with, alcohol marketing, and that this is associated with consumption and higher-risk drinking in current drinkers and susceptibility in never drinkers above and below the legal purchasing age. Further scrutiny and examination of the UK's self-regulatory approach, and viable alternatives, is needed to identify feasible, appropriate, and effectives means of reducing marketing exposure in young people.

For peer review only - http://bmjopen.bmj.com/site/about/guidelines.xhtml

Running head: Alcohol marketing and consumption in young people in the UK

Author contribution statement: LH, CT, and JV led on study design and data acquisition. LH, CT, JV were involved in design of the study tools with support from AMM. NC and AMM planned the analysis, and this was conducted by AMM. NC and AMM lead on interpretation of the results, with input from LH, CT, and JV. NC drafted the manuscript, with support from AMM on methods and results, and all authors provided feedback and approved the final version of the manuscript.

Acknowledgements: The authors thank YouGov for their assistance in preparing and managing delivery of the survey and data. We also thank NatCen Social Research for their contribution to the original scoping studies used in the development of this project.

Funding: This work was supported by a grant from Cancer Research UK (1107098).

Declaration of interest: Nathan Critchlow is a board member of Alcohol Focus Scotland. All other authors have no conflict of interest to declare.

Data sharing statement: No additional data are available.

For peer review only

Running head: Alcohol marketing and consumption in young people in the UK

REFERENCES

1. Newbury-Birch D, Walker J, Avery L, *et al*. *Impact of alcohol consumption on young people: A systematic review of published reviews,* London: Department for Children, Schools and Family, 2009.
2. McCambridge J, McAlaney K, Rowe R. Adult consequences of late adolescent alcohol consumption: A systematic review of cohort studies. *PloS Medicine* 2011;8(2):e1000413.
3. World Health Organisation. *Global status report on alcohol and health 2014.* Geneva: World Health Organisation, 2014.
4. National Health Service Digital. *Smoking, drinking, and drug use among young people: England, 2016.* London: Health and Social Care Information Service, 2017.
5. Public Health Wales Observatory. *Alcohol and health in Wales 2014: Wales profile.* Cardiff: Public Health Wales Observatory, 2014.
6. Scottish Government. *Scottish Schools Adolescent Lifestyle and Substance Use Survey (SALSUS): Alcohol report (2015).* Edinburgh: Scottish Government, 2016.
7. Public Health England. *The public health burden of alcohol and the effectiveness and cost-effectiveness of alcohol control policies: An evidence review.* London: Public Health England, 2016.
8. Hastings G. Alcohol marketing: Grooming the next generation. *BMJ;*346:f1227.
9. Alcohol Focus Scotland. Alcohol Focus Scotland. Glasgow: Alcohol Focus Scotland, 2017.
10. Dade P. *Drink talking: 100 years of alcohol advertising,* Middlesex: Middlesex University Press, 2008.
11. Diageo. *Annual report, 2017*. London: Diageo PLC, 2017.

For peer review only - http://bmjopen.bmj.com/site/about/guidelines.xhtml

12. Collin J, Hill SE, Smith KE. Merging alcohol giants threaten global health. *BMJ* 2015;17(351):h6087.
13. Purves, RI, Critchlow N, Stead M *et al.* Alcohol marketing during the UEFA EURO 2016 football tournament: A frequency analysis. *Int J Environ Res Public Health* 2017;14(7):e704.
14. Atkinson AM, Ross-Houle KM, Begley E, *et al*. An exploration of alcohol advertising on social networking sites: An analysis of content, interactions and young people's perspectives. *Add Res Theory* 2016;25(2)91-102.
15. King C, Sigel M, Ross CS, *et al*. Alcohol advertising in magazines and underage readership: Are underage youth disproportionality exposed? *Alcohol: Clin Exp Res* 2017;41(1):1775-1782.
16. Thompson KM. Addiction media: Substances on screen. *Child Adolesc Psychiatr Clin N Am* 2005;14:473-489.
17. Lowe E, Britton J, Cranwell J. Alcohol content in the 'hyper reality' MTV show 'Geordie Shore'. *Alcohol Alcohol* 2018;53(1):337-343.
18. Hastings G, Brooks O, Stead M, *et al. "They'll drink bucket loads of the stuff": An analysis of internal alcohol industry advertising documents.* London: Alcohol Education Research Council, 2010.
19. Rhoades E, Jernigan DH. Risky messages in alcohol advertising, 2003-2007: results from content analysis. *J Adolesc Health* 2013;52(1):1116-121.
20. Jones, SC, Hall S, Kypri K. Should I drink responsibly, safely or properly? Confusing messages about reducing alcohol-related harm. *PLoS One* 2017;12(9):e0184705.
21. Smith SW, Atkin CK, Roznowski J. Are "drink responsibly" alcohol campaigns strategically ambiguous? *Health Commun.* 2006;20(1):1-11.

For peer review only - http://bmjopen.bmj.com/site/about/guidelines.xhtml

22. Anderson P, de Bruijn A, Angus K, *et al.* Impact of alcohol advertising and media exposure on adolescent alcohol use: A systematic review of longitudinal studies. *Alcohol Alcohol* 2009;44(3):229-243.

23. Jernigan DH, Noel J, Landon J, *et al.* Alcohol marketing and youth alcohol consumption: A systematic review of longitudinal studies published since 2008. *Addiction* 2018;112(S1):7-20.

24. Austin EW, Knaus C. Predicting the potential for risky behaviour among those "too young" to drink as the result of appealing advertising. *J Health Commun.* 2000;5(1):13-27.

25. McClure AC, Stoolmiller M, Tanski SE, *et al.* Alcohol marketing receptivity, marketing-specific cognitions and underage binge drinking. *Alcohol Clin Exp Res* 2013;37(1):404-413.

26. Alcohol Health Alliance. *Health First: An evidence-based alcohol strategy for the UK.* Stirling: University of Stirling, 2013.

27. Gordon R, Harris F, MacKintosh AM, *et al.* Assessing the cumulative impact of alcohol marketing on young people's drinking: Cross-sectional data findings. *Addict Res Theory* 2011;19(1):66-75.

28. Gordon R., MacKintosh AM., Moodie C. The impact of alcohol marketing on youth drinking behaviour: A two stage cohort study. *Alcohol Alcohol* 2010;45(5):470-480.

29. YouGov. *Panel methodology.* Available from: https://yougov.co.uk/about/panel-methodology/ [accessed 6th June 2018].

30. Ford A, MacKintosh AM, Moodie C, *et al.* Cigarette pack design and adolescent smoking susceptibility: a cross-sectional survey. *BMJ Open* 2013;3:e003282.

For peer review only - http://bmjopen.bmj.com/site/about/guidelines.xhtml

31. Gallopel-Morvan K, Spilka S, Mutatayi C, *et al.* France's Évin law on the control of alcohol advertising: Content, effectiveness, and limitations. *Addiction* 2017;112(S1):86-93.

32. Research in Practice. *Scoring standardised measures – All measures and guidance.* Available from: https://www.rip.org.uk/resources/publications/practice-tools-and-guides/scoring-standardised-measures--all-measures-and-guidance-compilation-2015/ [accessed 6th June 2018].

33. Chester J, Montgomery K, Dorfman L. *Alcohol marketing in the digital age.* Berkley, CA: Berkley Media Studies Group, 2010.

34. Critchlow N, Moodie C, Bauld L, *et al.* Awareness of, and participation with, digital alcohol marketing, and the association with frequency of high episodic drinking among young adults. *Drugs* 2016;23(4):328-336.

35. Meier PS. Alcohol marketing research: The need for a new agenda. *Addiction* 2011;106(3):466-471.

36. Holmes J, Meier PS, Angus C, *et al.* Scotland's policy on minimum unit pricing for alcohol: The legal barriers are gone so what are the implications for implementation and evaluation? *Addiction* 2018;113(2):203-204.

37. Advertising Standards Authority. *Committee of Advertising Practitioners Code (Broadcast): 18, Alcohol,* London: Advertising Standards Authority, 2018.

38. Portman Group. *Code of practice on the naming, packaging, and promotion of alcoholic drinks* (5th Edition). London: The Portman Group, 2015.

39. Hastings G, Brooks O, Stead M, *et al.* (2010). Failure of self-regulation of UK alcohol advertising. *BMJ* 2010;*340:*b5650.

For peer review only - http://bmjopen.bmj.com/site/about/guidelines.xhtml

40. Noel JK, Babor TF, Does industry self-regulation protect young from exposure to alcohol marketing? A review of compliance and compliant studies. *Addiction* 2017;112(S1):51-56.

41. Alcohol Concern and Alcohol Research UK. *Fit for purpose?* An analysis of the role of the Portman Group in alcohol industry self-regulation. London: Alcohol Concern and Alcohol Research UK, 2018.

42. Fogarty AS, Chapman S. What should be done about policy on alcohol pricing and promotions? Australians experts' views of policy priorities: A qualitative interviews study. *BMC Public Health* 2013;13:610.

43. Morey Y, Eadie D, Purves RI, *et al*. *Specialist views of alcohol marketing regulation in the UK,* London: Cancer Research UK, 2017.

44. Morey Y, Eadie D, Purves RI, *et al*. *Youth engagement with alcohol brands in the UK,* London: Cancer Research UK, 2017.

45. Purves RI, Stead M, Eadie D. "I wouldn't be friends with some if they were liking too much rubbish": A qualitative study of alcohol brands, youth identify, and social media. *Int J Environ Res Public Health* 2018;15(2):e349.

46. Lin EY, Casswell S, You RQ, *et al.* Engagement alcohol marketing and early brand allegiance in relation to early years of drinking. *Add Res Theory* 2012;20(4):329-338.

For peer review only - http://bmjopen.bmj.com/site/about/guidelines.xhtml

Running head: Alcohol marketing and consumption in young people in the UK

Table 1. Awareness of alcohol marketing in the past month for young people in the UK

Marketing channel	Every day [28][1] %	5-6 times per week [22][1] %	3-4 times per week [14][1] %	1-2 times per week [6][1] %	Less than once a week [2][1] %	Not in the last month [0][1] %	Not sure %	Seen at least weekly %	Median Score (IQR)[2]
Adverts for alcohol									
… in newspapers or magazines	1.9	1.8	4.9	10.2	12.2	42.3	26.8	18.8	0 (6)
… on television	5.0	5.4	12.0	20.5	15.4	22.4	19.3	42.9	6 (14)
… on billboards	3.0	3.1	7.4	14.3	17.2	30.2	24.8	27.9	2 (6)
… on radio	1.1	1.0	2.3	5.1	7.1	54.7	28.8	9.4	0 (0)
… on YouTube, Tumblr, Facebook, Snapchat, Instagram or other social media	2.9	2.3	8.1	14.0	15.6	32.1	25.0	27.3	2 (6)
Famous people in films, music videos, on TV or pictured in magazines with alcohol	4.9	5.3	10.8	17.6	14.4	23.2	23.6	38.7	6 (14)
Sport sponsorship	2.4	3.4	7.9	17.0	17.4	27.8	24.1	30.7	2 (6)
Special offers	5.3	5.3	12.3	18.8	14.1	21.3	22.8	41.7	6 (14)
Competitions	1.4	1.2	2.8	8.2	11.9	45.6	29.0	13.6	0 (2)

Notes:
[1] Score for estimating the approximate number of days on which noticed alcohol marketing in a one month period.
[2] Median number of alcohol marketing instances noticed in a one month period.
Base: All respondents (n=3,399): weighted.

For peer review only - http://bmjopen.bmj.com/site/about/guidelines.xhtml

Running head: Alcohol marketing and consumption in young people in the UK

Table 2. Classification of alcohol marketing awareness (low, medium, and high) by demographic and confounding variables

Variable	*Valid n* (*n* = 1,411)[1]		Low awareness (score: ≤16)	Medium awareness (score: 17 -53)	High awareness (score: ≥ 54)	χ^2	*p*
**Gender**						9.26	<0.01
Male	735	%	32.1	30.5	37.4		
Female	676	%	37.3	32.8	29.9		
**Ethnicity**						1.09	n.s.
White British	1082	%	35.0	29.3	35.6		
Other ethnicity	317	%	34.5	32.3	33.3		
**IMD Quintile**						10.56	n.s.
1 (most deprived)	247	%	34.4	26.3	39.3		
2	266	%	35.7	28.2	36.1		
3	288	%	36.8	31.9	31.3		
4	292	%	32.2	34.6	33.2		
5 (least deprived)	317	%	34.1	35.3	30.6		
**Country lived in**						6.89	n.s.
England	1230	%	34.5	32.0	33.6		
Scotland	93	%	34.4	33.3	32.3		
Wales	53	%	39.6	30.2	30.2		
Northern Ireland	34	%	29.4	17.6	52.9		
**Legal purchase age**						14.10	<0.01
No	995	%	37.6	30.7	31.8		
Yes	416	%	27.4	33.7	38.9		
**Current drinker**						114.04	<0.001
No	609	%	49.9	26.9	23.2		
Yes	330	%	23.1	34.8	42.1		
**Higher risk drinker**						85.84	<0.001
No	1027	%	41.7	29.1	29.2		
Yes	384	%	15.6	38.3	46.1		
**Education**						13.90	<0.001
Not in education	79	%	17.7	31.6	50.6		
In education	1330	%	35.6	31.7	32.8		
**Working status**						7.93	<0.05
Not in work	1282	%	35.6	31.6	32.8		
In work	127	%	24.4	32.3	43.3		
**Parents accept use**						63.06	<0.001
No	722	%	44.2	28.4	27.4		
Yes	689	%	24.4	35.0	40.6		
**Peer accept use**							
No	410	%	51.5	24.4	24.1	73.08	<0.001
Yes	1001	%	27.7	34.6	37.8		

Notes: Valid sample refers to those for whom it was possible to compute an aggregate awareness score (i.e. excluding those who had reported 'not sure' to any marketing channels).
χ^2 = Bivariate Pearson Chi Square

For peer review only - http://bmjopen.bmj.com/site/about/guidelines.xhtml

Running head: Alcohol marketing and consumption in young people in the UK

For peer review only

For peer review only - http://bmjopen.bmj.com/site/about/guidelines.xhtml

Running head: Alcohol marketing and consumption in young people in the UK

Table 3. Association between alcohol marketing awareness and AUDIT-C scoring in current drinkers

	Unstandardized coefficients				Standard		
**Variables and reference categories**	***b***	**95% CI Low**	**95% CI Low**	**SE**	***β***	***t***	***p***
**Constant**	-5.57	-7.05	-4.09	0.75		-7.40	<0.001
**Age**	0.43	0.35	0.51	0.04	0.30	10.70	<0.001
**Gender**							
Male (*vs. female*)	0.31	0.09	0.54	0.11	0.06	2.76	0.006
**Ethnicity**							
White British *(vs. other)*	0.08	-0.24	0.40	0.16	0.01	0.52	n.s.
**IMD Quintile**							
(1 : most deprived to 5: most affluent)	0.11	0.03	0.20	0.04	0.06	2.68	0.007
**Country**							
Scotland (vs. England)	-0.05	-0.40	0.31	0.18	-0.01	-0.26	n.s.
Wales & Northern Ireland (vs. England)	-0.37	-0.76	0.01	0.20	-0.04	-1.90	n.s.
**Educational status**							
In education (*vs. not*)	0.66	0.55	1.10	0.22	0.07	2.96	0.003
**Working status**							
Working (*vs. not*)	0.31	-0.06	0.67	0.19	0.04	1.66	n.s.
**Living status**							
Living independently (*vs. with parents/adult family*)	0.87	0.54	1.20	0.17	0.12	5.17	<0.001
Not stated (*vs. with parents/adult family*)	0.42	-066	1.49	0.55	0.02	0.76	n.s.
**Frequency of mother drinking**							
Never (*vs. at least monthly*)	0.04	-0.41	0.49	0.23	0.00	0.17	n.s.
Less than monthly (*vs. at least monthly*)	-0.31	-0.63	0.00	0.16	-0.04	-1.94	n.s.
Not stated (*vs. at least monthly*)	0.42	-0.04	0.71	0.31	0.03	1.33	n.s.
**Frequency of father drinking**							
Never (*vs. at least monthly*)	0.21	-0.33	0.75	0.27	0.02	0.77	n.s.
Less than monthly (*vs. at least monthly*)	0.32	-0.08	0.72	0.20	0.03	1.57	n.s.
Not stated (*vs. at least monthly*)	0.33	-0.04	0.71	0.19	0.04	1.76	n.s.
**Frequency of close friends drinking**							
At least weekly (*vs. less often or never*)	1.44	1.19	1.69	0.13	0.26	11.32	<0.001
Not stated (*vs. less than weekly or never*)	-0.49	-0.85	-0.12	0.19	-0.06	-2.61	<0.01
**Parents' views**							
Drinking acceptable (*vs. neutral/unacceptable*)	0.29	0.01	0.57	0.14	0.05	2.06	<0.05
**Peer views**							

For peer review only

For peer review only - http://bmjopen.bmj.com/site/about/guidelines.xhtml

Drinking acceptable (*vs. neutral/unacceptable*)		0.08	-0.32	0.48	0.21		0.01		0.38	n.s.
**Age of first drink**										
Age 13 or under (*vs. 14 to 15 years*)		0.22	-0.07	0.51	0.15		0.04		1.50	n.s.
Age 16 or over (*vs. 14 to 15 years*)		-1.33	-1.63	-1.04	0.15		-0.21		-8.82	<0.001
Not stated (*vs. 14 to 15 years*)		-0.48	-0.89	-0.07	0.21		-0.05		-2.28	<0.05
**Alcohol Marketing Awareness**										
Medium (*vs. low awareness*)		0.79	0.37	1.21	0.21		0.11		3.70	<0.001
High (*vs. low awareness*)		0.85	0.44	1.26	0.21		0.12		4.08	<0.001
Not stated (*vs. low awareness*)		0.40	0.04	0.76	0.18		0.07		2.20	<0.05
**Own alcohol branded merchandise**										
Yes (*vs. no/not sure*)		0.79	0.55	1.04	0.13		0.13		6.30	<0.001

Notes:
Based on current drinkers: n = 1,592
DV = AUDIT-C Scoring (0-12)
Model shown is final block. Total variance explained (Adj. $R^2 = 0.36$). Durbin Watson = 2.01.
Final step model change: F (4, 1,564) = 17.44, $p<0.001$.
Overall Final model ANOVA: F (27, 1,564) = 34.34, $p<0.001$.

For peer review only

Running head: Alcohol marketing and consumption in young people in the UK

Table 4. Logistic regression of association between alcohol marketing and higher risk consumption among current drinkers

	Higher risk consumption among current drinkers				
	n	AOR[1]	95% CI	95% CI	*p*
**Age**	1,131	1.40	1.28	1.53	<0.001
**Gender**					
Female	824	Ref			
Male	768	1.32	1.04	1.68	0.022
**Ethnicity**					
Other	228	Ref			
White British	1,364	0.97	0.69	1.37	n.s.
**IMD Quintile**					n.s.
1 (most deprived)	232	Ref			
2	334	1.65	1.08	2.52	0.021
3	324	1.26	0.90	1.76	n.s.
4	340	1.21	0.90	1.64	n.s.
5 (most affluent)	362	1.23	0.93	1.64	n.s.
**Country**					n.s.
England	1,243	Ref			
Scotland	197	0.88	0.60	1.28	n.s.
Wales	116	0.58	0.36	0.93	0.024
Northern Ireland	36	1.35	0.60	3.01	n.s.
**Educational status**					
Not in education	146	Ref			
In education	1,446	1.61	1.01	2.55	0.045
**Working status**					
Not working	1,374	Ref			
Working (full or part-time)	218	1.43	0.97	2.09	n.s.
**Living status**					
Living with parents/adult family	1,307	Ref			
Living independently	268	1.56	1.09	2.23	0.015
Not stated	17	1.58	0.54	4.60	n.s.
**Frequency of mother drinking**					0.012
Never	115	ref			
Less than monthly vs. never	284	0.47	0.27	0.79	0.005
Monthly or fortnightly vs. less often	279	1.22	0.83	1.79	n.s.
At least weekly vs. less often	849	.93	.70	1.24	n.s.
Not stated vs. all other categories	65	1.50	.78	2.88	n.s.
**Frequency of father drinking**					n.s.
Never	76	ref			
Less than monthly vs. never	160	1.40	0.72	2.73	n.s.
Monthly or fortnightly vs. less often	201	0.73	0.46	1.19	n.s.
At least weekly vs. less often	964	0.83	0.61	1.15	n.s.

For peer review only - http://bmjopen.bmj.com/site/about/guidelines.xhtml

Running head: Alcohol marketing and consumption in young people in the UK

Not stated vs. all other categories	191	1.14	0.75	1.72	n.s.
**Frequency of close friends drinking**					<0.001
Never	72	ref			
Less than monthly vs. never	187	0.68	0.32	1.42	n.s.
Monthly or fortnightly vs. less often	463	2.20	1.44	3.35	<0.001
At least weekly vs. less often	667	3.41	2.48	4.70	<0.001
Not stated vs. all other categories	203	0.57	0.37	0.89	0.013
**Parents' views**					
Neutral or unacceptable	473	Ref			
Drinking acceptable	1,119	0.92	0.68	1.24	n.s.
**Peer views**					
Neutral or unacceptable	156	Ref			
Drinking acceptable	1,436	1.41	0.88	2.25	n.s.
**Age of first drink**					<0.001
Age 13 or under	472	Ref			
Age 14 to 15 (vs. 13 or under)	535	0.86	0.63	1.18	n.s.
Age 16 or over (vs. younger)	412	0.26	0.19	0.35	<0.001
Not stated	173	0.89	0.59	1.35	n.s.
**Alcohol Marketing Awareness**					<0.001
Low awareness	184	Ref			
Medium vs. low	274	2.18	1.39	3.42	<0.001
High vs. medium and low	326	1.43	1.01	2.02	0.45
Not stated vs. all other categories	808	0.85	0.67	1.08	n.s.
**Own alcohol branded merchandise**					
No or not sure	1,138	Ref			
Yes	454	1.71	1.31	2.22	<0.001

Notes:

Based on current drinkers (n = 1,592)

DV: Higher risk drinking on the AUDIT-C ($\geq$5), 1 = Higher risk (n = 699) and 0 = Lower risk (n = 893)

Test of model coefficients in final block: χ^2 (35) = 477.29, p<0.001.

Hosmer & Lemeshow for final block χ^2 (8) = 11.66, p = 0.17.

Nagelkerke R^2 for final block =0.35.

Cases correctly classified in final block: 72% in final block

[1] Adjusted for all other variables in the model, Adj OR, adjusted odds ratio; ref, reference category; 95% CI, 95% confidence interval

For peer review only

Running head: Alcohol marketing and consumption in young people in the UK

Table 5. Logistic regression of association between alcohol marketing and never drinkers' susceptibility to drink

	Susceptibility to drink among never drinkers				
	n	**Adj OR[1]**	**95% CI**	**95% CI**	***p***
**Age**	1580	1.05	0.98	1.13	n.s.
**Gender**					
Female	791	ref			
Male	789	1.09	0.88	1.37	n.s.
**Ethnicity**					
Other	377	ref			
White British	1203	1.51	1.12	2.03	0.007
**IMD Quintile**					n.s.
1 (most deprived)	399	ref			
2	278	1.13	0.80	1.60	n.s.
3	355	1.02	0.76	1.36	n.s.
4	233	0.88	0.64	1.22	n.s.
5 (most affluent)	315	0.84	0.63	1.11	n.s.
**Country**					n.s.
England	1193	ref			
Scotland	191	1.14	0.80	1.61	n.s.
Wales	115	1.09	0.70	1.69	n.s.
Northern Ireland	81	0.96	0.58	1.59	n.s.
**Educational status**					
Not in education	25	ref			
In education	1555	0.67	0.20	2.25	n.s.
**Working status**					
Not working	1550	ref			
Working (full or part-time)	30	2.59	0.83	8.11	n.s.
**Living status**					n.s.
Living with parents/adult family	1545	ref			
Living independently	28	0.51	0.20	1.28	n.s.
Not stated	7	1.57	0.27	9.11	n.s.
**Frequency of mother drinking**					<0.001
Never	321	ref			<0.001
Less than monthly vs. never	382	2.38	1.58	3.59	<0.001
Monthly or fortnightly vs. less often	242	1.66	1.15	2.39	0.006
At least weekly vs. less often	560	1.47	1.11	1.94	0.007
Not stated vs. all other categories	75	1.25	0.70	2.25	n.s.
**Frequency of father drinking**					0.023
Never	273	ref			
Less than monthly vs. never	217	1.88	1.17	3.01	0.009
Monthly or fortnightly vs. less often	232	1.11	0.75	1.64	n.s.
At least weekly vs. less often	686	1.39	1.05	1.84	0.021

For peer review only

Running head: Alcohol marketing and consumption in young people in the UK

Not stated vs. all other categories	172	1.06	0.71	1.58	n.s.
**Frequency of close friends drinking**					<0.001
Never	922	ref			
Less than monthly vs. never	162	3.46	2.26	5.26	<0.001
Monthly or fortnightly vs. less often	80	3.32	1.66	6.65	<0.001
At least weekly vs. less often	83	0.70	0.39	1.26	n.s.
Not stated vs. all other categories	333	0.61	0.43	0.86	0.005
**Parents' views**					
Neutral or unacceptable	1364	ref			
Drinking acceptable	216	1.00	0.70	1.44	n.s.
**Peer views**					
Neutral or unacceptable	894	ref			
Drinking acceptable	686	2.29	1.77	2.96	<0.001
**Alcohol Marketing Awareness**					n.s.
Low awareness	279	ref			
Medium vs. low	148	1.44	0.92	2.28	n.s.
High vs. medium and low	117	1.16	0.71	1.90	n.s.
Not stated vs. all other categories	1036	1.21	0.94	1.56	n.s.
**Own alcohol branded merchandise**					
No or not sure	1476	ref			
Yes	104	1.98	1.20	3.24	0.007

Notes:

Based on never drinkers (n = 1,580)

DV: Susceptibility: 1 = Susceptible (n = 830) = 0 Not susceptible (n = 750).

Test of model coefficients in final block: χ^2 (32) = 337.46, p<0.001.

Hosmer & Lemeshow for final block χ^2 (8) = 5.86, p <0.001

Nagelkerke R^2 for final block = 0.26.

Cases correctly classified in final block: 69%

[1] adjusted for all other variables in the model, Adj OR, adjusted odds ratio; ref, reference category; 95% CI, 95% confidence interval

[2] Variable not applicable to those who were classed as 'never drinkers'

For peer review only - http://bmjopen.bmj.com/site/about/guidelines.xhtml

STROBE 2007 (v4) Statement—Checklist of items that should be included in reports of *cross-sectional studies*

Section/Topic	Item #	Recommendation	Reported on page #
**Title and abstract**	1	(*a*) Indicate the study's design with a commonly used term in the title or the abstract	1,2,3
		(*b*) Provide in the abstract an informative and balanced summary of what was done and what was found	2
**Introduction**			
Background/rationale	2	Explain the scientific background and rationale for the investigation being reported	4,5,6
Objectives	3	State specific objectives, including any prespecified hypotheses	5,6
**Methods**			
Study design	4	Present key elements of study design early in the paper	6
Setting	5	Describe the setting, locations, and relevant dates, including periods of recruitment, exposure, follow-up, and data collection	6
Participants	6	(*a*) Give the eligibility criteria, and the sources and methods of selection of participants	6
Variables	7	Clearly define all outcomes, exposures, predictors, potential confounders, and effect modifiers. Give diagnostic criteria, if applicable	6,7,8,9
Data sources/ measurement	8*	For each variable of interest, give sources of data and details of methods of assessment (measurement). Describe comparability of assessment methods if there is more than one group	6,7,8,9
Bias	9	Describe any efforts to address potential sources of bias	N/A
Study size	10	Explain how the study size was arrived at	N/A
Quantitative variables	11	Explain how quantitative variables were handled in the analyses. If applicable, describe which groupings were chosen and why	9,10,11
Statistical methods	12	(*a*) Describe all statistical methods, including those used to control for confounding	9,10,11
		(*b*) Describe any methods used to examine subgroups and interactions	9,10,11
		(*c*) Explain how missing data were addressed	9,10,11
		(*d*) If applicable, describe analytical methods taking account of sampling strategy	9,10,11
		(*e*) Describe any sensitivity analyses	N/A
**Results**			

For peer review only

Participants	13*	(a) Report numbers of individuals at each stage of study—eg numbers potentially eligible, examined for eligibility, confirmed eligible, included in the study, completing follow-up, and analysed	11,12,13,14
		(b) Give reasons for non-participation at each stage	13,14
		(c) Consider use of a flow diagram	N/A
Descriptive data	14*	(a) Give characteristics of study participants (eg demographic, clinical, social) and information on exposures and potential confounders	11
		(b) Indicate number of participants with missing data for each variable of interest	11,12,13
Outcome data	15*	Report numbers of outcome events or summary measures	11,12,13,14,15
Main results	16	(*a*) Give unadjusted estimates and, if applicable, confounder-adjusted estimates and their precision (eg, 95% confidence interval). Make clear which confounders were adjusted for and why they were included	11,12,13,14,15
		(*b*) Report category boundaries when continuous variables were categorized	11,12,13,14,15
		(*c*) If relevant, consider translating estimates of relative risk into absolute risk for a meaningful time period	11,12
Other analyses	17	Report other analyses done—eg analyses of subgroups and interactions, and sensitivity analyses	N/A
**Discussion**			
Key results	18	Summarise key results with reference to study objectives	15,18
Limitations	19	Discuss limitations of the study, taking into account sources of potential bias or imprecision. Discuss both direction and magnitude of any potential bias	17,18
Interpretation	20	Give a cautious overall interpretation of results considering objectives, limitations, multiplicity of analyses, results from similar studies, and other relevant evidence	15,16,17,18
Generalisability	21	Discuss the generalisability (external validity) of the study results	15,16,17,18
**Other information**			
Funding	22	Give the source of funding and the role of the funders for the present study and, if applicable, for the original study on which the present article is based	19

*Give information separately for cases and controls in case-control studies and, if applicable, for exposed and unexposed groups in cohort and cross-sectional studies.

Note: An Explanation and Elaboration article discusses each checklist item and gives methodological background and published examples of transparent reporting. The STROBE checklist is best used in conjunction with this article (freely available on the Web sites of PLoS Medicine at http://www.plosmedicine.org/, Annals of Internal Medicine at http://www.annals.org/, and Epidemiology at http://www.epidem.com/). Information on the STROBE Initiative is available at www.strobe-statement.org.

For peer review only

BMJ Open

Awareness of alcohol marketing, ownership of alcohol branded merchandise, and the association with alcohol consumption, higher-risk drinking, and drinking susceptibility in adolescents and young adults: A cross-sectional survey in the United Kingdom.

Journal:	*BMJ Open*
Manuscript ID	bmjopen-2018-025297.R1
Article Type:	Research
Date Submitted by the Author:	12-Nov-2018
Complete List of Authors:	Critchlow, Nathan; University of Stirling, Institute for Social Marketing<br>Mackintosh, Anne Marie; University of Stirling Institute for Social Marketing<br>Thomas, Christopher; Cancer Research UK, Cancer Policy Research Centre<br>Hooper, Lucie; Cancer Research UK, Cancer Policy Research Centre<br>Vohra, Jyotsna; Cancer Research UK, Cancer Policy Research Centre
**Primary Subject Heading**:	Public health
Secondary Subject Heading:	Addiction, Public health
Keywords:	PUBLIC HEALTH, Alcohol, Young people, Alcohol Marketing, Survey, Alcohol Advertising

SCHOLARONE™
Manuscripts

Running head: Alcohol marketing and consumption in young people in the UK

Title: Awareness of alcohol marketing, ownership of alcohol branded merchandise, and the association with alcohol consumption, higher-risk drinking, and drinking susceptibility in adolescents and young adults: A cross-sectional survey in the United Kingdom.

Running head: Alcohol marketing and consumption in young people in the UK

Authors

Nathan Critchlow[1]

Anne Marie MacKintosh[1]

Christopher Thomas[2]

Lucie Hooper[2]

Jyotsna Vohra[2]

[1] Institute for Social Marketing, Faculty of Health Sciences and Sport, University of Stirling, Stirling.

[2] Cancer Policy Research Centre (CPRC), Cancer Research UK, London.

Corresponding author

Nathan Critchlow, Institute for Social Marketing, Faculty of Health Sciences and Sport, University of Stirling, Stirling, FK9 4LA. E-mail: nathan.critchlow@stir.ac.uk; Tel: +44(0)1786467382.

Word Count: 4,491

Table and figure count: 6 (All tables)

For peer review only - http://bmjopen.bmj.com/site/about/guidelines.xhtml

Running head: Alcohol marketing and consumption in young people in the UK

Awareness of alcohol marketing, ownership of alcohol branded merchandise, and the association with alcohol consumption, higher-risk drinking, and drinking susceptibility in adolescents and young adults: A cross-sectional survey in the United Kingdom.

ABSTRACT

Objectives: To explore awareness of alcohol marketing and ownership of alcohol branded merchandise in adolescents and young adults in the United Kingdom (UK), what factors are associated with awareness and ownership, and what association awareness and ownership has with alcohol consumption, higher-risk drinking, and susceptibility.

Design: Online cross-sectional survey conducted April–May 2017.

Setting: UK.

Participants: Adolescents and young adults aged 11-19 years old in the UK (*n*=3,399).

Main outcome measures: Alcohol Use Disorders Identification Test–Consumption (AUDIT-C) (0-12) and indication of higher-risk consumption ($\geq$5 AUDIT-C) in current drinkers. Susceptibility to drink in the next year (Yes/No) in never drinkers.

Results: Eighty-two percent of respondents were aware of at least one form of alcohol marketing in the past month and 17% owned branded merchandise. Chi-square tests found that higher awareness of marketing and ownership of branded merchandise was associated with being a current drinker (p<0.001), higher-risk drinking (p<0.001), frequency of parental and peer consumption (p<0.001), and perceived parental (p<0.001) and peer approval of consumption (p<0.001). Among current drinkers, multivariate regressions (controlling for demographics and covariates) found that marketing awareness and owning branded merchandise was positively associated with AUDIT-C score and higher-risk consumption. For example, current drinkers reporting medium marketing awareness were twice as likely to be higher-risk drinkers as those reporting low awareness (AOR=2.18, 95% CI: 1.39-3.42, p<0.001). Among never drinkers, respondents who owned branded merchandise were twice as likely to be susceptible to drinking as those who did not (AOR=1.98, 95% CI: 1.20-3.24, p=0.007).

Conclusions: Young people, above and below the legal purchasing age, are aware of alcohol marketing through a range of channels and almost one-in-five own alcohol branded merchandise. In current drinkers, alcohol marketing awareness was associated with increased consumption and greater likelihood of higher-risk consumption. In never drinkers, ownership of branded merchandise was associated with susceptibility.

For peer review only - http://bmjopen.bmj.com/site/about/guidelines.xhtml

Running head: Alcohol marketing and consumption in young people in the UK

Keywords: Alcohol marketing; Young people; Higher-risk drinking; Alcohol Advertising; Susceptibility; Survey; Public Health; Health Policy.

Strengths and limitations of this study

- This is the first study to examine awareness of alcohol marketing and ownership of alcohol branded merchandise in a demographically representative sample of young people across the UK, including those above and below the legal purchasing age for alcohol.
- The study provides timely insight into what forms of alcohol marketing young people are aware of, how frequently they recall seeing alcohol marketing, and what factors are associated with higher awareness of alcohol marketing and ownership of alcohol branded merchandise.
- The large sample size supports robust statistical analysis to examine what relationship (if any) there is between alcohol marketing and consumption, controlling for demography and relevant covariates (e.g. peer consumption).
- The study explores the association between alcohol marketing and consumption at three levels: overall alcohol consumption and higher-risk drinking in current drinkers, and susceptibility in never-drinkers.
- The cross-sectional nature of the survey does not enable causal relationships to be drawn about the link between alcohol marketing and either consumption or susceptibility.

For peer review only

Running head: Alcohol marketing and consumption in young people in the UK

Awareness of alcohol marketing, ownership of alcohol branded merchandise, and the association with alcohol consumption, higher-risk drinking, and drinking susceptibility in adolescents and young adults: A cross-sectional survey in the United Kingdom.

INTRODUCTION

Adolescents and young adults (hereafter 'young people', aged 11- 19 years old) are a focal population for alcohol research because consumption at this stage of development is associated with increased drinking and risk of concomitant harms in later adulthood [1,2]. Global estimates indicate that consumption by young people is particularly high in Europe, where the proportion of current drinkers (69.5%) is higher than the five other global regions, and the proportion of lifetime abstainers is lower (15.9%) [3]. In England, it is estimated that approximately half of 11-15-year olds (44%) have consumed an alcoholic drink, one-in-ten have consumed in the past week, and nine percent have been drunk in the past month [4]. Similar estimates are reported in Scotland and Wales [5,6]. Understanding the drivers of alcohol consumption in young people is important given the immediate and long-term individual, social, and economic consequences associated with higher-risk drinking [7].

One factor routinely cited as shaping alcohol-related attitudes and behaviours in young people is marketing [8,9]. Marketing is fundamentally important to alcohol producers. It represents the primary method of communicating with new and existing consumers, can directly encourage sales, and can increase brand salience over competitors. Accordingly, alcohol companies have used highly visible marketing for over 100 years [10], with the current UK landscape characterised by a complex network of mass media marketing (e.g. television), alternative marketing (e.g. sponsorship), consumer marketing (e.g. price), and stakeholder marketing (e.g. to retailers) [9]. The importance of marketing to the alcohol industry is evidenced through their annual investment, with Diageo's global marketing expenditure approximately £1.8 billion [11]. Continued consolidation in the alcohol industry has also seen

For peer review only

For peer review only - http://bmjopen.bmj.com/site/about/guidelines.xhtml

the market become dominated by a small number of transnational producers, creating larger marketing budgets, economies of scale, and intense competition [12].

Content analysis research, which focuses on the marketing output as the unit of analysis, consistently reports that marketing may reach and influence young people. For example, marketing has been reported in media environments where young people may be exposed, including sports [13], social media [14], print media [15], and on-screen [16, 17]. Content research has also found that marketing may appeal to young people through creative designs, use of topical and real-world associations which may resonate with younger audiences, and by promoting positive connotations around consumption (e.g. sociability or desirable lifestyles) [18,19]. It has also been suggested that commercial marketing contains ambiguous messages about lower-risk consumption [20,21].

Systematic reviews of consumer research, which focus on the individual as the unit of analysis, provide consistent evidence that awareness of, and participation with, marketing has a causal influence on young people's consumption, including initiation and frequency of drinking [22,23]. Qualitative research has also suggested that this relationship is more complex than an 'exposure equals consumption' hypothesis, and that young people consider alcohol marketing and branding to hold rich cultural, social, and symbolic meaning [14,24,25]. Accordingly, message interpretation research has attempted to move the debate on from whether marketing is associated with consumption and onto how this influence occurs, by identifying psychological mechanisms which mediate the relationship between exposure and consumption [26,27].

In the UK, the influence of alcohol marketing on young people has been a topic of debate for decades [9,28]. These debates are further supplemented by concerns about the efficacy and effectiveness of self-regulation, the predominant approach employed to control alcohol marketing in the UK. This includes suggestions that self-regulation provides inadequate

For peer review only - http://bmjopen.bmj.com/site/about/guidelines.xhtml

Running head: Alcohol marketing and consumption in young people in the UK

restrictions, is not consistently enforced or complied with, is retrospective and slow to react to complaints, lacks meaningful sanctions, and lags behind modern marketing methods [9,28-32]. There are, however, unresolved issues which have inhibited attempts to move the debate forward. In the UK, the last large-scale assessment of young people's awareness of alcohol marketing is a decade old, was only conducted in Scotland, only sampled adolescents under the minimum purchase age, only considered overall marketing awareness (not frequency), and did not consider higher-risk consumption [33,34].

In this study, we explore frequency of awareness for alcohol marketing and ownership of alcohol branded merchandise in a demographically representative sample of young people in the UK, including those above and below the legal purchasing age. We also consider what association (if any) awareness of alcohol marketing and ownership of branded merchandise has with alcohol consumption and higher-risk drinking in current drinkers, and susceptibility to drink in never drinkers.

METHOD

Design and sample

Data come from the 2017 Youth Alcohol Policy Survey, an online cross-sectional survey conducted with 11-19-year olds in the UK (n=3,399). Responses were collected April–May 2017. The survey was hosted by YouGov, a market research company, who recruited a sample intended to be representative of the UK population from their UK panel [35]. Participants aged 16 or over were approached directly to participate, while those aged under 16 were approached through existing adult panel members known to have children. A survey weight was provided for each respondent (based on age, gender, ethnicity, region, and social grade) to enable descriptive data to be representative of the UK population. The study design was informed by

For peer review only - http://bmjopen.bmj.com/site/about/guidelines.xhtml

previous cross-sectional surveys in the UK which have explored young people's experiences of alcohol and tobacco marketing [33,36].

Measures

Demography

Alcohol consumption is not homogeneous among young people in the UK [4-7]. It is therefore important to adjust for demographic variation when examining any factors purported to be associated with consumption. In this study, age, gender, ethnicity, resident country (England, Scotland, Wales, Northern Ireland), living status, employment status, educational status, legal purchasing status for alcohol (≥18 years old), and indices of deprivation (IMD), were obtained from information held about panel respondents or survey questions.

Awareness of alcohol marketing

Awareness of alcohol marketing was assessed through structured, self-reported recall, a method frequently used in consumer research [33]. Participants were prompted with the statement '*Over the last month, how often, if at all, have you seen…*' and then presented with descriptions of nine examples of alcohol marketing: (1) newspapers or magazines; (2) television; (3) billboards; (4) radio; (5) adverts on social media (e.g. YouTube, Tumblr, Facebook, SnapChat, Instagram or other social media); (6) famous people in films, music videos or TV or pictured in magazines with alcohol [celebrity endorsement]; (7) sports, games, or events sponsorship; (8) special price offers; and (9) competitions or prize draws. As per recent research [37,38], a Likert scale was used to measure frequency of noticing marketing in the past month for each of the nine examples (1=Everyday – 6=Not in the past month; Not sure).

In the UK, survey research which has measured awareness of alcohol marketing has typically used dichotomous response options for each channel (e.g. Yes/No) and used a

For peer review only - http://bmjopen.bmj.com/site/about/guidelines.xhtml

summation across these to estimate overall awareness. [33,39]. This method, however, only provides insight into breadth of marketing awareness across channels, not frequency or volume, and therefore lacks sensitivity and may underestimate awareness. To enhance accuracy in this study, the self-reported frequency of awareness for each marketing example was converted into the estimated number of days that marketing had been seen in a four-week period (i.e. 'one month'). This timeframe is consistent with previous research [40,41] and is representative of the minimum number of days in any month. For example, an answer of 'everyday' equated to 28 instances of awareness over four weeks (i.e. seven days per-week multiplied by four) and 1-2 times per-week equated to six instances over four weeks (i.e. 1.5 times per-week multiplied by four) (see Table 1 for other response options). Scores across the nine channels were summed to create an aggregate score, providing an approximation of total alcohol marketing awareness in the past month. Estimating total volume of awareness, as opposed to breadth across channels, is consistent with other recent alcohol marketing research [42,43].

In this study, an aggregate awareness score was only computed when a valid answer had been given for all nine marketing examples. To provide meaningful interpretative utility, the aggregate score for the valid sample was split into tertiles of low (aggregate score ≤16; awareness approximately every other day), medium (17-53; awareness approximately daily), and high awareness (≥54; awareness almost twice daily). If a participant answered 'not sure' to any of the nine channels they were coded as 'not stated' for the aggregate score. Indicating 'not sure' meant that a respondent's potential aggregate score was, by default, more conservative than those who provided a valid answer to all nine examples. These respondents were therefore coded as a separate 'not sure' category to avoid biasing the proportion of valid respondents considered to have low or medium awareness, or what the tertiles boundaries were.

Ownership of alcohol brand merchandise

For peer review only - http://bmjopen.bmj.com/site/about/guidelines.xhtml

Running head: Alcohol marketing and consumption in young people in the UK

Ownership of alcohol branded merchandise was measured through a single item adapted from previous research [33,44]. Participants were asked '*Do you own any merchandise (such as clothing or drinks glasses) that show an alcoholic drink brand or logo?*' (Yes/No/Not sure).

Alcohol consumption status

Participants were asked '*Have you ever had a whole alcoholic drink? Not just a sip*?' [33,34]. Those who answered 'No' were classed as never-drinkers while those who answered 'Yes' were classed as ever-drinkers.

Alcohol consumption and higher-risk drinking

Alcohol use was measured through the Alcohol Use Disorders Identification Test–Consumption (AUDIT-C), which assessed frequency of consumption, units drunk in a typical drinking occasion, and frequency of heavy episodic drinking. Responses were provided on five-point scales, with the answers for each item relative to frequency (0=Never – 4= Four or more times a week), units drunk (0=1-2 units – 4=10 units or more), or frequency of heavy episodic drinking (0=Never – 4=Daily or almost Daily). Heavy episodic drinking was classified as eight or more units in a single occasion for males, and six or more units for females (one unit=8g or 10ml of alcohol). A diagram depicting the unit content of alcoholic drinks was included to assist calculation of units. Those who answered anything other than 'never' on the first AUDIT-C item were classed as current drinkers and asked to complete the final two items. All other respondents (i.e. those stating 'never' for frequency of consumption) were classified as non-drinkers and were not asked to complete the final two items. In current drinkers, a total AUDIT-C score was computed by summing the three AUDIT-C items (0-12), with a cut-off of ≥5 used to identify higher-risk consumption [45].

For peer review only - http://bmjopen.bmj.com/site/about/guidelines.xhtml

Running head: Alcohol marketing and consumption in young people in the UK

Susceptibility

As per tobacco research, susceptibility was defined as the absence of a firm decision not to drink alcohol in the next year [36]. Never-drinkers were classified as 'non-susceptible' if they answered 'definitely no' to the question '*Do you think you will drink alcohol at any time during the next year*?' Those who answered anything other than 'Definitely no' were classified as Susceptible.

Confounding variables

Confounding factors, reported to influence consumption in young people and used in previous alcohol marketing research, were included as covariates to contextualise any association between marketing and consumption [33,34,46,47]. Frequency of consumption was measured for the mother (female carer), father (male carer), and closest friend (each scored: 1=Never – 9=Every day or almost every day; Prefer not to say; Not applicable). For all three groups, consumption was collapsed into five categories (Never, Less than monthly, Monthly or Fortnightly, At Least weekly, and Not Stated). Perceived acceptability of consumption was measured for parents and peers (each scored: 1=Total acceptable – 5=Totally unacceptable). For both groups, acceptability was converted into dichotomous categories ('Neutral or unacceptable' and 'Acceptable'). For ever drinkers, age of first drink was also measured (≤8 years old – 19 years old; Can't remember; Prefer not to say). Answers were converted into three categories (≤13 years; 14-15 years; ≥16 years).

Ethics

Ethical approval was obtained from the University of Stirling's General University Ethics Panel (GUEP59). YouGov included a lead for ethical and quality assurance, including consent,

For peer review only - http://bmjopen.bmj.com/site/about/guidelines.xhtml

post-survey debriefing and signposting to support organisations, and confidentiality and anonymity.

Patient and Public Involvement

The survey was developed following cognitive testing with a small sample (*n*=100) of young people to ensure age and cultural comprehension of the questions. Beyond this, no other patient or public involvement was undertaken.

Analysis

Data were analysed using SPSS version 23 (SPSS Inc., Chicago IL). Descriptive data were weighted so that percentages and median scores were representative of the demographic profile of the UK population. Bivariate analyses, using Chi-square tests, examined differences in level of alcohol marketing awareness and ownership of branded merchandise between the demographic and confounding variables.

A multivariate linear regression was conducted with current drinkers' AUDIT-C score as the dependent variable (0-12) and awareness of marketing and ownership of branded merchandise as the key independent variables. The following demographic and confounding variables were also included in initial blocks: age; gender; ethnicity; IMD quintile; resident country; educational status; working status; living status; frequency of mother (female carer), father (male carer) and close friend drinking; perceived parental and peer acceptability of consumption; and drink age of first drink. Categorical variables with ≥3 categories were converted into dummy (binary) variables to aid interpretation and comparison. The omitted dummy variable formed the reference category. For example, marketing awareness was a categorical variable with four levels: low, medium, high, and not stated. Four binary variables were computed: low awareness, medium awareness, high awareness, and not stated (each

For peer review only

For peer review only - http://bmjopen.bmj.com/site/about/guidelines.xhtml

coded Yes=1, No=0). By including medium, high and not stated in the multivariate analysis, and omitting low awareness, the reference category was low awareness. The multivariate regressions therefore indicate the association between level of consumption and medium awareness, relative to low awareness, and high awareness relative to low awareness. Reference categories for each variable are displayed in the results.

Two multivariate logistic regressions were conducted with higher-risk drinking (AUDIT-C ≥5) among current drinkers and susceptibility to drink among never-drinkers as the dependent variables. Marketing awareness and ownership of branded merchandise were the key independent variables. Where applicable, both logistic regressions controlled for the same demographic and confounding variables as the linear regression. Reference categories for categorical independent variables are indicated in the results. Where the categorical variables had three ≥3 levels, and were of an ordinal level, the SPSS contrast=difference function enabled comparison of each increasing category relative to the combined previous categories. For example, the first comparison with frequency of mother's drinking and higher-risk drinking was 'less than monthly drinking' vs. 'mother never drinks', whereas the final comparison was 'at least weekly drinking' vs. 'less often'. As the independent variables were categorical, 'not stated' responses were also included as a separate category and compared against the reference category for each variable. This enabled the maximum sample to be retained. For example, the large number of 'not stated' responses on level of marketing awareness could be compared with those for whom marketing awareness could be computed.

All multivariate analyses were conducted on unweighted data as the factors used to construct the weights were included as covariates in the models. The multivariate analyses were repeated on weighted data to check for consistency. As results for the key independent variables (marketing awareness and ownership of branded merchandise) were consistent, only the unweighted results are presented.

For peer review only - http://bmjopen.bmj.com/site/about/guidelines.xhtml

Running head: Alcohol marketing and consumption in young people in the UK

RESULTS

Sample characteristics

The weighted sample (*n*=3,399) had an average age of 15.18 years old (*SD*=2.55; range: 11-19), with three quarters (76%) below the legal purchasing age (≤18 years). There was an even distribution for gender (51% male and 49% female). The majority of the sample were White British (76%) and were evenly distributed across IMD (20% in each quintile). Most participants lived in England (84%) with the remainder from Scotland (8%), Wales (5%), and Northern Ireland (3%). Almost all participants were living at home with parent(s) or other adult family members (90%) and were in some form of education (95%).

Alcohol consumption and susceptibility

After excluding cases with missing data on drinking status (*n*=62, weighted), almost half of the weighted sample (48%; *n*=1,590) were current drinkers. Within current drinkers, the average AUDIT-C score was 4.33 (*SD*=2.77). Almost half of current drinkers (44%; *n*=707) were classified as higher-risk (≥5 AUDIT-C). After excluding cases with missing data on drinking status (*n*=62, weighted), almost half of the weighted sample (49%; *n*=1,623) were never drinkers. Within never drinkers, half were classified as susceptible (52%; *n*=841).

Awareness of alcohol marketing

The most frequent sources of marketing awareness in the past month were adverts on television (Median 6 instances per month, Inter quartile range=14), celebrity endorsement (Median 6, IQR=14), and special offers (Median 6, IQR=14) (Table 1). More than a third of respondents (range: 39-43%) had noticed marketing through these channels at least weekly. Billboard adverts (Median 2 instances per month, IQR=6), sponsorship (Median 2, IQR=6), and social

For peer review only - http://bmjopen.bmj.com/site/about/guidelines.xhtml

media adverts (Median 2, IQR = 6) were noticed less than once a week, with at least a quarter of participants (range: 27-31%) having noticed these at least weekly. Lowest awareness was for adverts in the print press (Median 0 instances per month, IQR=6), on radio (Median 0, IQR = 0), and competitions (Median 0, IQR=2). For each marketing example, a fifth or more (range: 19-29%) were not sure how often, if at all, they had come across alcohol marketing. Overall, 82% had noticed marketing through at least one channel.

[TABLE 1]

Aggregate alcohol marketing awareness

The median aggregate alcohol marketing awareness score was 32 (IQR=60), equating to noticing 32 instances of alcohol marketing in the past month (under minimum purchase age: median=28; IQR=60). When categorised into tertiles, 35% of the valid sample were classified as having low awareness (≤16 instances per month), 32% had medium awareness (17–53), and 34% had high awareness (≥54). In those under the minimum purchase age, 38% had low awareness, 31% medium, and 32% high.

Bivariate Chi-square tests found that higher awareness of alcohol marketing was significantly associated with being male, of legal purchasing age, a current drinker, a higher-risk drinker, not in education, in employment, and perceiving that parents and peers would consider it okay to consume (Table 2). Higher awareness was also associated with greater frequency of mother (female carer) consumption, $\chi^2(16)=38.25$, $p<0.001$, greater frequency of father (male carer) consumption, $\chi^2(16)=29.55$, $p<0.05$, and greater frequency of close friends drinking, $\chi^2(16)=198.51$, $p<0.001$. There was no difference in awareness by ethnicity, IMD quintile, or resident country.

For peer review only - http://bmjopen.bmj.com/site/about/guidelines.xhtml

[TABLE 2]

Owning alcohol branded merchandise

Almost a fifth of participants (17%) reported owning alcohol branded merchandise. Bivariate Chi-square tests found that ownership of branded merchandise was significantly associated with being of white British ethnicity, of legal purchase age, a current drinker, a higher-risk drinker, not in education, in employment, and perceiving that parents and peers would consider it okay to consume (Table 3). Ownership of branded merchandise was also associated with greater frequency of mother (female carer) consumption, $\chi^2(8)=44.11$, $p<0.001$, greater frequency of father (male carer) consumption, $\chi^2(8)=56.49$, $p<0.001$, and greater frequency of close friends drinking, $\chi^2(8)=178.76$, $p<0.001$. There was also an overall effect of IMD, $\chi^2(4)=15.73$, $p<0.01$, although this had no distinct pattern across escalating deprivation. There was no difference by resident country or gender.

[TABLE 3]

Association between alcohol marketing and AUDIT-C scoring

A multivariate linear regression examined the association between marketing awareness, ownership of branded merchandise, and AUDIT-C scoring in current drinkers (Table 4). After controlling for demographic and confounding factors, medium alcohol marketing awareness ($b=0.79$, 95% CI: 0.37-1.21, $p<0.001$), or high awareness ($b=0.85$, 95% CI: 0.44-1.26, $p<0.001$), compared to low awareness, was associated with higher AUDIT-C score, as was ownership of branded merchandise ($b=0.79$, 95% CI: 0.55-1.04, $p<0.001$). Of the demographic variables, being older ($p<0.001$), male ($p=0.006$), from a more affluent IMD ($p<0.01$), in education ($p<0.01$), and living independently of parents or adult family members ($p<0.001$)

For peer review only - http://bmjopen.bmj.com/site/about/guidelines.xhtml

was also associated with higher AUDIT-C score in the final model. Of the confounding variables, having a close friend who drinks at least weekly ($p<0.001$), and perceiving that parents consider it acceptable to consume ($p<0.05$) was also associated with higher AUDIT-C score. Having a first alcoholic drink at ≥16 years old ($p<0.001$) was associated with lower AUDIT-C score, compared with those who first drank aged 14–15 years.

[TABLE 4]

Association between alcohol marketing and higher-risk consumption

A multivariate logistic regression examined the association between marketing awareness, ownership of branded merchandise, and higher-risk drinking in current drinkers (Table 5). After controlling for demographic and confounding factors, medium alcohol marketing awareness (Adjusted Odds Ratio=2.18, 95% CI: 1.39-3.42, $p<0.001$), high awareness (AOR=1.43, 95% CI: 1.01-2.02, $p<0.05$), and owning branded merchandise (AOR=1.71, 95% CI: 1.31-2.22, $p<0.001$) were associated with higher-risk drinking. Of the demographic variables, being older ($p<0.001$), male ($p<0.05$), from England compared to Wales ($p<0.05$), in education ($p<0.05$), and living independently ($p<0.05$) was associated with higher-risk drinking in the final model. Of the confounding variables, increasing frequency of close friend consumption ($p<0.001$), and having had first drink aged 14-15 years old or younger ($p<0.001$), was associated with higher-risk consumption.

[TABLE 5]

Association between alcohol marketing and susceptibility to consume

For peer review only - http://bmjopen.bmj.com/site/about/guidelines.xhtml

Running head: Alcohol marketing and consumption in young people in the UK

A multivariate logistic regression examined the association between marketing awareness, ownership of branded merchandise, and susceptibility to drink in never drinkers (Table 6). After controlling for demographic and confounding variables, awareness of alcohol marketing was not associated with susceptibility, but ownership of branded merchandise was, with those who owned branded merchandise almost twice as likely to be susceptible compared to those who did not (AOR=1.98, 95% CI: 1.20-3.24, $p<0.01$). Of the demographic variables, only being white British ($p<0.01$) was associated with susceptibility in the final model. Of the confounding variables, frequency of mother (female carer) consumption ($p<0.001$), frequency of father (male carer) consumption ($p<0.05$), frequency of close friend consumption ($p<0.001$), and perceived peer approval ($p<0.001$) were associated with susceptibility.

[TABLE 6]

DISCUSSION

The findings indicate that young people in the UK are aware of a variety of alcohol marketing and almost a fifth own branded merchandise. The results also show that awareness of marketing and ownership of branded merchandise is associated with increased consumption and higher-risk drinking in current drinkers, and that ownership of branded merchandise is associated with susceptibility in never-drinkers. We address key evidence gaps in the UK by exploring frequency of marketing awareness (not just breadth of exposure) and demonstrating an association between marketing and both consumption and susceptibility in young people above and below the legal purchasing age from across the UK.

The findings are consistent with suggestions that alcohol marketing appears in contexts which may reach young people, including those under the legal purchasing age [8,9]. Awareness included mass media marketing (e.g. television), alternative marketing (e.g.

For peer review only - http://bmjopen.bmj.com/site/about/guidelines.xhtml

sponsorship and celebrity endorsement), consumer marketing (e.g. price offers), and digital media. This highlights the dynamic nature of '360-degree' marketing strategies and how they reach young people in offline and online environments [9,48]. The results extend understanding by showing how frequently young people see alcohol marketing; with at least one-in-ten reporting daily or almost daily awareness through three of the nine marketing examples. Approximately half of the sample had seen at least 32 instances of alcohol marketing per month, which equates to awareness at least once a day. Although there were expected differences in awareness between drinkers and never-drinkers [33,49], there were no differences between key demographic groups, including ethnicity, indices of deprivation, and resident country. This suggests that awareness of alcohol marketing occurs in young people across the UK, and is not isolated to a minority of demographic groups.

The results are consistent with longitudinal research which has shown a link between marketing and increased consumption in young people [22,23,34,38]. Although marketing awareness did not have an association with susceptibility in never-drinkers, ownership of branded merchandise did. Research has reported that participation with marketing has a stronger association with consumption than awareness [33,34,39,49]. Our findings therefore suggest that the effect of participation is pronounced in never-drinkers. Nevertheless, as research suggests that not all alcohol marketing or brands are equally appealing to youth [25,50], it is possible that focusing on aggregated alcohol marketing awareness (the approach in this study) may have disguised associations between individual examples of marketing and susceptibility in never drinkers. The findings also extend understanding by showing an association between marketing and consumption across young adulthood. This includes an association with susceptibility and consumption in young people under the legal purchasing age and higher-risk drinking in newly-legal drinkers. Newly legal drinkers are an important target for alcohol marketers [18] and are a key under-researched group [51]. The findings

For peer review only - http://bmjopen.bmj.com/site/about/guidelines.xhtml

Running head: Alcohol marketing and consumption in young people in the UK

therefore highlight the importance of considering the wider role that marketing plays on consumption, not just in those under the purchasing age.

Except for the Scottish Government's decision to implement minimum unit pricing [52], there has been little recent change to the UK's self-and-co-regulatory framework for alcohol marketing [53,54]. It is claimed that such self-regulatory approaches provide inadequate restrictions, are not suitably enforced, are retrospective and slow to react to complaints, and lack meaningful sanctions [9,28-32]. Although statutory regulations are cited as an alternative approach [28], studies have also questioned whether current examples, such as the Loi Évin in France, are being enforced properly or whether they reduce marketing exposure [13,37]. Further research exploring the perspectives of stakeholders involved in the production, research, consumption, and regulation of marketing would be of value to identify feasible and effective options to reduce youth exposure and form a consensus on appropriate action [55,56].

There are limitations. First, the cross-sectional design cannot identify a causal relationship between marketing and consumption, albeit a directional effect is supported by longitudinal research [22,23]. Moreover, that marketing had any association with consumption and susceptibility at all suggests that it must at least play either an initiating or reinforcing role. Second, the results are only partially representative of young adults above the legal purchasing age, albeit other research has shown similar trends in older young adults [39]. Third, the marketing channels measured are not exhaustive and, consequently, the results may underestimate awareness. Examples of omitted marketing include packaging, cinema, product placement, and a broader range of digital marketing [33,39,49]. It was also not possible to decipher whether 'not sure' responses indicated uncertainty over whether a participant had seen alcohol marketed at all through a channel or uncertainty on the frequency of awareness. This influenced the design of the regression models (to account for a 'Not sure' category). Third,

For peer review only - http://bmjopen.bmj.com/site/about/guidelines.xhtml

except for owning branded merchandise, the study only measured awareness of marketing, but not participation. As participation is reported to have a stronger effect [34,49], the results may underestimate the association between marketing and drinking outcomes. Finally, measurement of owning branded merchandise also only included two examples as prompts (clothing and drinks glasses). It is possible that different prompts may have altered recall, and that multiple items or a free text response option would have provided greater clarity on merchandise owned.

CONCLUSION

This paper makes important contributions to understanding by exploring awareness of alcohol marketing and ownership of branded merchandise by young people from across the UK, three quarters of who were under the legal purchasing age. The results highlight that '360-degree' marketing strategies have created several avenues for young people to be exposed to, or involved with, alcohol marketing, and that this is associated with consumption and higher-risk drinking in current drinkers and susceptibility in never drinkers. Further scrutiny and examination of the UK's self-regulatory approach, and viable alternatives, is needed to identify feasible, appropriate, and effective means of reducing marketing exposure in young people.

Author contribution statement: LH, CT, and JV led on study design and data acquisition. LH, CT, JV were involved in design of the study tools with support from AMM. NC and AMM planned the analysis, and this was conducted by AMM. NC and AMM led on interpretation of the results, with input from LH, CT, and JV. NC drafted the manuscript, with support from AMM on methods and results, and all authors provided feedback and approved the final version of the manuscript.

For peer review only - http://bmjopen.bmj.com/site/about/guidelines.xhtml

Running head: Alcohol marketing and consumption in young people in the UK

Acknowledgements: The authors thank YouGov for their assistance in preparing and managing delivery of the survey and data. We thank Gillian Rosenberg from the CPRC at Cancer Research UK for her role in managing the project and providing information on YouGov fieldwork. We also thank NatCen Social Research for their contribution to the original scoping studies used in the development of this project.

Funding: This work was supported by a grant from Cancer Research UK (1107098).

Declaration of interest: Nathan Critchlow is a board member of Alcohol Focus Scotland. All other authors have no conflict of interest to declare.

Data sharing statement: No additional data are available.

For peer review only

Running head: Alcohol marketing and consumption in young people in the UK

REFERENCES

1. Newbury-Birch D, Walker J, Avery L, *et al*. *Impact of alcohol consumption on young people: A systematic review of published reviews,* London: Department for Children, Schools and Family, 2009.
2. McCambridge J, McAlaney K, Rowe R. Adult consequences of late adolescent alcohol consumption: A systematic review of cohort studies. *PloS Medicine* 2011;8(2):e1000413.
3. World Health Organisation. *Global status report on alcohol and health 2014.* Geneva: World Health Organisation, 2014.
4. National Health Service Digital. *Smoking, drinking, and drug use among young people: England, 2016.* London: Health and Social Care Information Service, 2017.
5. Public Health Wales Observatory. *Alcohol and health in Wales 2014: Wales profile.* Cardiff: Public Health Wales Observatory, 2014.
6. Scottish Government. *Scottish Schools Adolescent Lifestyle and Substance Use Survey (SALSUS): Alcohol report (2015).* Edinburgh: Scottish Government, 2016.
7. Public Health England. *The public health burden of alcohol and the effectiveness and cost-effectiveness of alcohol control policies: An evidence review.* London: Public Health England, 2016.
8. Hastings G. Alcohol marketing: Grooming the next generation. *BMJ;*346:f1227.
9. Alcohol Focus Scotland. Alcohol Focus Scotland. Glasgow: Alcohol Focus Scotland, 2017.
10. Dade P. *Drink talking: 100 years of alcohol advertising,* Middlesex: Middlesex University Press, 2008.
11. Diageo. *Annual report, 2017*. London: Diageo PLC, 2017.

For peer review only - http://bmjopen.bmj.com/site/about/guidelines.xhtml

12. Collin J, Hill SE, Smith KE. Merging alcohol giants threaten global health. *BMJ* 2015;17(351):h6087.
13. Purves, RI, Critchlow N, Stead M *et al.* Alcohol marketing during the UEFA EURO 2016 football tournament: A frequency analysis. *Int J Environ Res Public Health* 2017;14(7):e704.
14. Atkinson AM, Ross-Houle KM, Begley E, *et al*. An exploration of alcohol advertising on social networking sites: An analysis of content, interactions and young people's perspectives. *Add Res Theory* 2016;25(2)91-102.
15. King C, Sigel M, Ross CS, *et al*. Alcohol advertising in magazines and underage readership: Are underage youth disproportionality exposed? *Alcohol: Clin Exp Res* 2017;41(1):1775-1782.
16. Thompson KM. Addiction media: Substances on screen. *Child Adolesc Psychiatr Clin N Am* 2005;14:473-489.
17. Lowe E, Britton J, Cranwell J. Alcohol content in the 'hyper reality' MTV show 'Geordie Shore'. *Alcohol Alcohol* 2018;53(1):337-343.
18. Hastings G, Brooks O, Stead M, *et al. "They'll drink bucket loads of the stuff": An analysis of internal alcohol industry advertising documents.* London: Alcohol Education Research Council, 2010.
19. Rhoades E, Jernigan DH. Risky messages in alcohol advertising, 2003-2007: results from content analysis. *J Adolesc Health* 2013;52(1):1116-121.
20. Jones, SC, Hall S, Kypri K. Should I drink responsibly, safely or properly? Confusing messages about reducing alcohol-related harm. *PLoS One* 2017;12(9):e0184705.
21. Smith SW, Atkin CK, Roznowski J. Are "drink responsibly" alcohol campaigns strategically ambiguous? *Health Commun.* 2006;20(1):1-11.

For peer review only - http://bmjopen.bmj.com/site/about/guidelines.xhtml

Running head: Alcohol marketing and consumption in young people in the UK

22. Anderson P, de Bruijn A, Angus K, *et al.* Impact of alcohol advertising and media exposure on adolescent alcohol use: A systematic review of longitudinal studies. *Alcohol Alcohol* 2009;44(3):229-243.
23. Jernigan DH, Noel J, Landon J, *et al.* Alcohol marketing and youth alcohol consumption: A systematic review of longitudinal studies published since 2008. *Addiction* 2018;112(S1):7-20.
24. Morey Y, Eadie D, Purves RI, *et al*. *Youth engagement with alcohol brands in the UK,* London: Cancer Research UK, 2017.
25. Purves RI, Stead M, Eadie D. "I wouldn't be friends with some if they were liking too much rubbish": A qualitative study of alcohol brands, youth identify, and social media. *Int J Environ Res Public Health* 2018;15(2):e349.
26. Austin EW, Knaus C. Predicting the potential for risky behaviour among those "too young" to drink as the result of appealing advertising. *J Health Commun.* 2000;5(1):13-27.
27. McClure AC, Stoolmiller M, Tanski SE, *et al.* Alcohol marketing receptivity, marketing-specific cognitions and underage binge drinking. *Alcohol Clin Exp Res* 2013;37(1):404-413.
28. Alcohol Health Alliance. *Health First: An evidence-based alcohol strategy for the UK.* Stirling: University of Stirling, 2013.
29. Noel JK, Babor TF, Does industry self-regulation protect young from exposure to alcohol marketing? A review of compliance and compliant studies. *Addiction* 2017;112(S1):51-56.
30. Noel JK, Babor TF, Robaina K. Industry self-regulation of alcohol marketing: A systematic review of content and exposure research. *Addiction* 2017; 112(S1):28-50.

For peer review only - http://bmjopen.bmj.com/site/about/guidelines.xhtml

31. Alcohol Concern and Alcohol Research UK. *Fit for purpose?* An analysis of the role of the Portman Group in alcohol industry self-regulation. London: Alcohol Concern and Alcohol Research UK, 2018.

32. Hastings G, Brooks O, Stead M, *et al.* (2010). Failure of self-regulation of UK alcohol advertising. *BMJ* 2010;*340:*b5650.

33. Gordon R, Harris F, MacKintosh AM, *et al.* Assessing the cumulative impact of alcohol marketing on young people's drinking: Cross-sectional data findings. *Addict Res Theory* 2011;19(1):66-75.

34. Gordon R., MacKintosh AM., Moodie C. The impact of alcohol marketing on youth drinking behaviour: A two stage cohort study. *Alcohol Alcohol* 2010;45(5):470-480.

35. YouGov. *Panel methodology*. Available from: https://yougov.co.uk/about/panel-methodology/ [accessed 6th June 2018].

36. Ford A, MacKintosh AM, Moodie C, *et al.* Cigarette pack design and adolescent smoking susceptibility: a cross-sectional survey. *BMJ Open* 2013;3:e003282.

37. Gallopel-Morvan K, Spilka S, Mutatayi C, *et al.* France's Évin law on the control of alcohol advertising: Content, effectiveness, and limitations. *Addiction* 2017;112(S1):86-93.

38. de Bruijn A, Tanghe J, de Leeuw R, *et al.* European longitudinal study on the relationship between adolescents' alcohol marketing exposure and alcohol use. *Addiction* 2016*;*111(10):17774-1783.

39. Critchlow N, Moodie C, Bauld L, *et al.* Awareness of, and participation with, digital alcohol marketing, and the association with frequency of high episodic drinking among young adults. *Drugs* 2016;23(4):328-336.

40. Synder LB, Fleming F, Slater M, *et al.* Effects of alcohol advertising exposure on drinking among youth. *Arch Pediatr Adolesc Med.* 2006;160(1):18-24.

For peer review only - http://bmjopen.bmj.com/site/about/guidelines.xhtml

Running head: Alcohol marketing and consumption in young people in the UK

41. Siegel M, Kurland RP, Castrini M, *et al.* Potential youth exposure to alcohol advertising on the internet: A study of internet version of popular television programmes. *J Subs Use* 2016;21(4): 361-367.

42. Collins RL, Martino SC, Kovalchik SA, *et al.* Alcohol advertising exposure among middle school-age youth: An assessment across all media and venues. *J Stud Alcohol Drugs* 2016;77(3):384-392.

43. Chambers T, Stanley J, Signal L, *et al.* Quantifying the nature and extent of children's real-time exposure to alcohol marketing in their everyday lives using wearable cameras: Children's exposure via a range of media in a range of key places. *Alcohol Alcohol* 2018;53(5):626-633.

44. Jones SC. Alcohol-branded merchandise ownership and drinking. *Pediatrics* 2016;137(5):1-12.

45. Research in Practice. *Scoring standardised measures – All measures and guidance.* Available from: https://www.rip.org.uk/resources/publications/practice-tools-and-guides/scoring-standardised-measures--all-measures-and-guidance-compilation-2015/ [accessed 6th June 2018].

46. Rossow I, Keating P, Felix L, McCambridge J. Does parental drinking influence children's drinking? A systematic review of prospective cohort studies. *Addiction* 2016;111(2):204-217.

47. Leung RK, Toumbourou JW, Hemphill SA. The effect of peer influence and selection processes on adolescent alcohol use: A systematic review of longitudinal studies. *Health Psychol Rev* 2014;8(4):426-457.

48. Chester J, Montgomery K, Dorfman L. *Alcohol marketing in the digital age.* Berkley, CA: Berkley Media Studies Group, 2010.

For peer review only - http://bmjopen.bmj.com/site/about/guidelines.xhtml

Running head: Alcohol marketing and consumption in young people in the UK

49. Lin E-Y, Casswell S, You RQ, Huckle T. Engagement with alcohol marketing and early brand allegiance in relation to early years of drinking. *Addict Res Theory* 2012;20(4):329-338.
50. Padon AA, Rimal, DeJong W, *et al.* Assessing youth-appealing content in alcohol advertisements: Application of a content appealing to youth (CAY) index. *Health Commun.* 2018;32(2):164-173.
51. Meier PS. Alcohol marketing research: The need for a new agenda. *Addiction* 2011;106(3):466-471.
52. Holmes J, Meier PS, Angus C, *et al.* Scotland's policy on minimum unit pricing for alcohol: The legal barriers are gone so what are the implications for implementation and evaluation? *Addiction* 2018;113(2):203-204.
53. Advertising Standards Authority. *Committee of Advertising Practitioners Code (Broadcast): 18, Alcohol,* London: Advertising Standards Authority, 2018.
54. Portman Group. *Code of practice on the naming, packaging, and promotion of alcoholic drinks* (5th Edition). London: The Portman Group, 2015.
55. Fogarty AS, Chapman S. What should be done about policy on alcohol pricing and promotions? Australians experts' views of policy priorities: A qualitative interviews study. *BMC Public Health* 2013;13:610.
56. Morey Y, Eadie D, Purves RI, *et al*. *Specialist views of alcohol marketing regulation in the UK,* London: Cancer Research UK, 2017.

For peer review only - http://bmjopen.bmj.com/site/about/guidelines.xhtml

Running head: Alcohol marketing and consumption in young people in the UK

Table 1. Awareness of alcohol marketing in the past month for young people in the UK

	Every day [28][1]	5-6 times per week [22][1]	3-4 times per week [14][1]	1-2 times per week [6][1]	Less than once a week [2][1]	Not in the last month [0][1]	Not sure	Seen at least weekly	Median Score (IQR)[2]
**Marketing channel**	%	%	%	%	%	%	%	%	
Adverts for alcohol									
… in newspapers or magazines	1.9	1.8	4.9	10.2	12.2	42.3	26.8	18.8	0 (6)
… on television	5.0	5.4	12.0	20.5	15.4	22.4	19.3	42.9	6 (14)
… on billboards	3.0	3.1	7.4	14.3	17.2	30.2	24.8	27.9	2 (6)
… on radio	1.1	1.0	2.3	5.1	7.1	54.7	28.8	9.4	0 (0)
… on YouTube, Tumblr, Facebook, Snapchat, Instagram or other social media	2.9	2.3	8.1	14.0	15.6	32.1	25.0	27.3	2 (6)
Famous people in films, music videos, on TV or pictured in magazines with alcohol	4.9	5.3	10.8	17.6	14.4	23.2	23.6	38.7	6 (14)
Sport sponsorship	2.4	3.4	7.9	17.0	17.4	27.8	24.1	30.7	2 (6)
Special offers	5.3	5.3	12.3	18.8	14.1	21.3	22.8	41.7	6 (14)
Competitions	1.4	1.2	2.8	8.2	11.9	45.6	29.0	13.6	0 (2)

Notes:
[1] Score for estimating the approximate number of days on which noticed alcohol marketing in a one month period.
[2] Median number of alcohol marketing instances noticed in a one month period.
Base: All respondents (n=3,399): weighted.

For peer review only - http://bmjopen.bmj.com/site/about/guidelines.xhtml

Running head: Alcohol marketing and consumption in young people in the UK

Table 2. Classification of alcohol marketing awareness (low, medium, and high) by demographic and confounding variables

Variable	*Valid n* (*n*= 1,411)[1]		Low awareness[2]	Medium awareness[3]	High awareness[4]	χ^2	*p*
**Gender**						9.26	<0.01
Male	735	%	32.1	30.5	37.4		
Female	676	%	37.3	32.8	29.9		
**Ethnicity**						1.09	n.s.
White British	1,082	%	34.5	32.3	33.3		
Other ethnicity	317	%	35.0	29.3	35.6		
**IMD Quintile**						10.56	n.s.
1 (most deprived)	247	%	34.4	26.3	39.3		
2	266	%	35.7	28.2	36.1		
3	288	%	36.8	31.9	31.2		
4	292	%	32.2	34.6	33.2		
5 (least deprived)	317	%	34.1	35.3	30.6		
**Country lived in**						6.89	n.s.
England	1,230	%	34.5	32.0	33.6		
Scotland	93	%	34.4	33.3	32.3		
Wales	53	%	39.6	30.2	30.2		
Northern Ireland	34	%	29.4	17.6	52.9		
**Legal purchase age**						14.10	<0.01
No	995	%	37.6	30.7	31.8		
Yes	416	%	27.4	33.7	38.9		
**Current drinker**						114.04	<0.001
No	609	%	49.9	26.9	23.2		
Yes	784	%	23.1	34.8	42.1		
**Higher risk drinker**						85.84	<0.001
No	1,027	%	41.7	29.1	29.2		
Yes	384	%	15.6	38.3	46.1		
**Education**						13.90	<0.001
Not in education	79	%	17.7	31.6	50.6		
In education	1,330	%	35.6	31.7	32.8		
**Working status**						7.93	<0.05
Not in work	1,282	%	35.6	31.6	32.8		
In work	127	%	24.4	32.3	43.3		
**Parents accept use**						63.06	<0.001
No	722	%	44.2	28.4	27.4		
Yes	689	%	24.4	35.0	40.6		
**Peer accept use**							
No	410	%	51.5	24.4	24.1	73.08	<0.001
Yes	1001	%	27.7	34.6	37.8		

Notes:
[1]Valid sample excludes those who had reported 'not sure' to any marketing channels); sample is weighted.
[2] Low awareness equals ≤16 instances per month (i.e. once every other day);
[3] Medium awareness equals 17-53 instances per month (i.e. almost once a day or more);
[4] High awareness equals ≥54 instances per month (i.e. almost twice a day or more);
χ^2 = Bivariate Pearson Chi Square.
Due to a large number of categories, analysis of how awareness of alcohol marketing varied by mother (female carer), father (male carer), and close friend frequency consumption only reported in text.

For peer review only

For peer review only - http://bmjopen.bmj.com/site/about/guidelines.xhtml

Running head: Alcohol marketing and consumption in young people in the UK

Table 3. Ownership of alcohol branded items by demographic and confounding variables

Variable	*Valid n* (*n* = 3,276)[1]		Own branded merchandise	χ^2	*p*
**Gender**				2.71	n.s.
Male	1,679	%	18.5		
Female	1,597	%	16.3		
**Ethnicity**				16.68	<0.001
White British	2,506	%	19.0		
Other ethnicity	745	%	12.5		
**IMD Quintile**				15.73	<0.01
1 (Most deprived)	652	%	13.5		
2	646	%	21.1		
3	644	%	17.2		
4	662	%	19.5		
5 (Least deprived)	655	%	16.0		
**Country lived in**				0.97	n.s.
England	2,759	%	17.4		
Scotland	260	%	16.2		
Wales	155	%	16.8		
Northern Ireland	103	%	20.4		
**Legal purchase age**				100.33	<0.001
No	2,488	%	13.7		
Yes	788	%	29.2		
**Current drinker**				256.07	<0.001
No	1,683	%	7.2		
Yes	1549	%	28.7		
**Higher risk drinker**				222.98	<0.001
No	2,543	%	12.3		
Yes	690	%	36.7		
**Education**				43.73	<0.001
Not in education	161	%	36.6		
In education	3,106	%	16.4		
**Working status**				31.08	<0.001
Not in work	3,028	%	16.3		
In work	239	%	30.5		
**Parents accept use**				189.06	<0.001
No	1,920	%	9.7		
Yes	1,357	%	28.2		
**Peer accept use**					
No	1,066	%	8.0	97.68	<0.001
Yes	2,210	%	21.9		

Notes:
[1]Valid sample refers to those who answered 'yes' or 'no'. Missing cases due to 'don't know' response (*n* = 123). Sample is weighted.
χ^2 = Bivariate Pearson Chi Square.
Due to a large number of categories, analysis of how ownership of alcohol branded merchandise varied by mother (female carer), father (male carer), and close friend frequency consumption only reported in text.

For peer review only

Running head: Alcohol marketing and consumption in young people in the UK

For peer review only

For peer review only - http://bmjopen.bmj.com/site/about/guidelines.xhtml

Running head: Alcohol marketing and consumption in young people in the UK

Table 4. Association between alcohol marketing awareness and AUDIT-C scoring in current drinkers

	**Unstandardized coefficients**				**Standard coefficient**		
**Variables and reference categories**	***b***	**95% CI Lower**	**95% CI Upper**	**SE**	***β***	***t***	***p***
**Constant**	-5.57	-7.05	-4.09	0.75		-7.40	<0.001
**Age**	0.43	0.35	0.51	0.04	0.30	10.70	<0.001
**Gender**							
Male (*vs. female*)	0.31	0.09	0.54	0.11	0.06	2.76	<0.01
**Ethnicity**							
White British *(vs. other)*	0.08	-0.24	0.40	0.16	0.01	0.52	n.s.
**IMD Quintile**							
(1 : most deprived to 5: most affluent)	0.11	0.03	0.20	0.04	0.06	2.68	<0.01
**Country**							
Scotland (vs. England)	-0.05	-0.40	0.31	0.18	-0.01	-0.26	n.s.
Wales & Northern Ireland (vs. England)	-0.37	-0.76	0.01	0.20	-0.04	-1.90	n.s.
**Educational status**							
In education (*vs. not*)	0.66	0.22	1.10	0.22	0.07	2.96	<0.01
**Working status**							
Working (*vs. not*)	0.31	-0.06	0.67	0.19	0.04	1.66	n.s.
**Living status**							
Living independently (*vs. with parents/adult family*)	0.87	0.54	1.20	0.17	0.12	5.17	<0.001
Not stated (*vs. with parents/adult family*)	0.42	-066	1.49	0.55	0.02	0.76	n.s.
**Frequency of mother drinking**							
Never (*vs. at least monthly*)	0.04	-0.41	0.49	0.23	0.00	0.17	n.s.
Less than monthly (*vs. at least monthly*)	-0.31	-0.63	0.00	0.16	-0.04	-1.94	n.s.
Not stated (*vs. at least monthly*)	0.42	-0.20	1.03	0.31	0.03	1.33	n.s.
**Frequency of father drinking**							
Never (*vs. at least monthly*)	0.21	-0.33	0.75	0.27	0.02	0.77	n.s.
Less than monthly (*vs. at least monthly*)	0.32	-0.08	0.72	0.20	0.03	1.57	n.s.
Not stated (*vs. at least monthly*)	0.33	-0.04	0.71	0.19	0.04	1.76	n.s.
**Frequency of close friends drinking**							
At least weekly (*vs. less often or never*)	1.44	1.19	1.69	0.13	0.26	11.32	<0.001
Not stated (*vs. less than weekly or never*)	-0.49	-0.85	-0.12	0.19	-0.06	-2.61	<0.01
**Parents' views**							
Drinking acceptable (*vs. neutral/unacceptable*)	0.29	0.01	0.57	0.14	0.05	2.06	<0.05
**Peer views**							

For peer review only

Drinking acceptable (*vs. neutral/unacceptable*)		0.08	-0.32	0.48	0.21		0.01		0.38	n.s.
**Age of first drink**										
Age 13 or under (*vs. 14 to 15 years*)		0.22	-0.07	0.51	0.15		0.04		1.50	n.s.
Age 16 or over (*vs. 14 to 15 years*)		-1.33	-1.63	-1.04	0.15		-0.21		-8.82	<0.001
Not stated (*vs. 14 to 15 years*)		-0.48	-0.89	-0.07	0.21		-0.05		-2.28	<0.05
**Alcohol Marketing Awareness**										
Medium (*vs. low awareness*)		0.79	0.37	1.21	0.21		0.11		3.70	<0.001
High (*vs. low awareness*)		0.85	0.44	1.26	0.21		0.12		4.08	<0.001
Not stated (*vs. low awareness*)		0.40	0.04	0.76	0.18		0.07		2.20	<0.05
**Own alcohol branded merchandise**										
Yes (*vs. no/not sure*)		0.79	0.55	1.04	0.13		0.13		6.30	<0.001

Notes:
Based on current drinkers: n = 1,592; data are unweighted.
DV = AUDIT-C Scoring (0-12)
Model shown is final block. Total variance explained (Adj. R^2 = 0.36). Durbin Watson = 2.01.
Final step model change: F (4, 1,564) = 17.44, p<0.001.
Overall Final model ANOVA: F (27, 1,564) = 34.33, p<0.001.

For peer review only

For peer review only - http://bmjopen.bmj.com/site/about/guidelines.xhtml

Running head: Alcohol marketing and consumption in young people in the UK

Table 5. Logistic regression of association between alcohol marketing and higher risk consumption among current drinkers

	Higher risk consumption among current drinkers				
	n	AOR[1]	95% CI Lower	95% CI Upper	*p*
**Age**	1,131	1.40	1.28	1.53	<0.001
**Gender**					
Female	824	Ref			
Male	768	1.32	1.04	1.68	<0.05
**Ethnicity**					
Other	228	Ref			
White British	1,364	0.97	0.69	1.37	n.s.
**IMD Quintile**					n.s.
1 (most deprived)	232	Ref			
2 vs. 1	334	1.65	1.08	2.52	<0.05
3 vs. 1,2	324	1.26	0.90	1.76	n.s.
4 vs. 1,2,3	340	1.21	0.90	1.64	n.s.
5 (most affluent) vs. 1,2,3,4	362	1.23	0.93	1.64	n.s.
**Country**					n.s.
England	1,243	Ref			
Scotland	197	0.88	0.60	1.28	n.s.
Wales	116	0.58	0.36	0.93	<0.05
Northern Ireland	36	1.35	0.60	3.01	n.s.
**Educational status**					
Not in education	146	Ref			
In education	1,446	1.61	1.01	2.55	<0.05
**Working status**					
Not working	1,374	Ref			
Working (full or part-time)	218	1.43	0.97	2.09	n.s.
**Living status**					
Living with parents/adult family	1,307	Ref			
Living independently	268	1.56	1.09	2.23	<0.05
Not stated	17	1.58	0.54	4.60	n.s.
**Frequency of mother drinking**					<0.05
Never	115	ref			
Less than monthly vs. never	284	0.47	0.27	0.79	<0.01
Monthly or fortnightly vs. less often	279	1.22	0.83	1.79	n.s.
At least weekly vs. less often	849	.93	.70	1.24	n.s.
Not stated vs. all other categories	65	1.50	.78	2.88	n.s.
**Frequency of father drinking**					n.s.
Never	76	ref			
Less than monthly vs. never	160	1.40	0.72	2.73	n.s.

For peer review only - http://bmjopen.bmj.com/site/about/guidelines.xhtml

Running head: Alcohol marketing and consumption in young people in the UK

Monthly or fortnightly vs. less often	201	0.73	0.46	1.19	n.s.
At least weekly vs. less often	964	0.83	0.61	1.15	n.s.
Not stated vs. all other categories	191	1.14	0.75	1.72	n.s.
**Frequency of close friends drinking**					<0.001
Never	72	ref			
Less than monthly vs. never	187	0.68	0.32	1.42	n.s.
Monthly or fortnightly vs. less often	463	2.20	1.44	3.35	<0.001
At least weekly vs. less often	667	3.41	2.48	4.70	<0.001
Not stated vs. all other categories	203	0.57	0.37	0.89	0.013
**Parents' views**					
Neutral or unacceptable	473	Ref			
Drinking acceptable	1,119	0.92	0.68	1.24	n.s.
**Peer views**					
Neutral or unacceptable	156	Ref			
Drinking acceptable	1,436	1.41	0.88	2.25	n.s.
**Age of first drink**					<0.001
Age 13 or under	472	Ref			
Age 14 to 15 (vs. 13 or under)	535	0.86	0.63	1.18	n.s.
Age 16 or over (vs. younger)	412	0.26	0.19	0.35	<0.001
Not stated	173	0.89	0.59	1.35	n.s.
**Alcohol Marketing Awareness**					<0.001
Low awareness	184	Ref			
Medium vs. low	274	2.18	1.39	3.42	<0.001
High vs. medium and low	326	1.43	1.01	2.02	<0.01
Not stated vs. all other categories	808	0.85	0.67	1.08	n.s.
**Own alcohol branded merchandise**					
No or not sure	1,138	Ref			
Yes	454	1.71	1.31	2.22	<0.001

Notes:
Based on current drinkers (n = 1,592); data are unweighted.
DV: Higher risk drinking on the AUDIT-C (≥5), 1 = Higher risk (n = 699) and 0 = Lower risk (n = 893)
Test of model coefficients in final block: χ^2 (35) = 477.29, p<0.001.
Hosmer & Lemeshow for final block χ^2 (8) = 11.66, p = 0.17.
Nagelkerke R^2 for final block =0.35.
Cases correctly classified in final block: 72% in final block
[1] Adjusted for all other variables in the model, Adj OR, adjusted odds ratio; ref, reference category; 95% CI, 95% confidence interval

For peer review only - http://bmjopen.bmj.com/site/about/guidelines.xhtml

Running head: Alcohol marketing and consumption in young people in the UK

Table 6. Logistic regression of association between alcohol marketing and never drinkers' susceptibility to drink

	Susceptibility to drink among never drinkers				
	n	**AOR[1]**	**95% CI Lower**	**95% CI Upper**	***p***
**Age**	1,580	1.05	0.98	1.13	n.s.
**Gender**					
Female	791	ref			
Male	789	1.09	0.88	1.37	n.s.
**Ethnicity**					
Other	377	ref			
White British	1,203	1.51	1.12	2.03	<0.01
**IMD Quintile**					n.s.
1 (most deprived)	399	ref			
2 vs. 1	278	1.13	0.80	1.60	n.s.
3 vs. 1,2	355	1.02	0.76	1.36	n.s.
4 vs. 1,2,3	233	0.88	0.64	1.22	n.s.
5 (most affluent) vs. 1,2,3,4	315	0.84	0.63	1.11	n.s.
**Country**					n.s.
England	1,193	ref			
Scotland	191	1.14	0.80	1.61	n.s.
Wales	115	1.09	0.70	1.69	n.s.
Northern Ireland	81	0.96	0.58	1.59	n.s.
**Educational status**					
Not in education	25	ref			
In education	1,555	0.67	0.20	2.25	n.s.
**Working status**					
Not working	1,550	ref			
Working (full or part-time)	30	2.59	0.83	8.11	n.s.
**Living status**					n.s.
Living with parents/adult family	1,545	ref			
Living independently	28	0.51	0.20	1.28	n.s.
Not stated	7	1.57	0.27	9.11	n.s.
**Frequency of mother drinking**					<0.001
Never	321	ref			<0.001
Less than monthly vs. never	382	2.38	1.58	3.59	<0.001
Monthly or fortnightly vs. less often	242	1.66	1.15	2.39	<0.01
At least weekly vs. less often	560	1.47	1.11	1.94	<0.01
Not stated vs. all other categories	75	1.25	0.70	2.25	n.s.
**Frequency of father drinking**					<0.05
Never	273	ref			
Less than monthly vs. never	217	1.88	1.17	3.01	<0.01
Monthly or fortnightly vs. less often	232	1.11	0.75	1.64	n.s.

For peer review only - http://bmjopen.bmj.com/site/about/guidelines.xhtml

For peer review only

Running head: Alcohol marketing and consumption in young people in the UK

At least weekly vs. less often	686	1.39	1.05	1.84	<0.05
Not stated vs. all other categories	172	1.06	0.71	1.58	n.s.
**Frequency of close friends drinking**					<0.001
Never	922	ref			
Less than monthly vs. never	162	3.46	2.26	5.26	<0.001
Monthly or fortnightly vs. less often	80	3.32	1.66	6.65	<0.001
At least weekly vs. less often	83	0.70	0.39	1.26	n.s.
Not stated vs. all other categories	333	0.61	0.43	0.86	<0.01
**Parents' views**					
Neutral or unacceptable	1,364	ref			
Drinking acceptable	216	1.00	0.70	1.44	n.s.
**Peer views**					
Neutral or unacceptable	894	ref			
Drinking acceptable	686	2.29	1.77	2.96	<0.001
**Alcohol Marketing Awareness**					n.s.
Low awareness	279	ref			
Medium vs. low	148	1.44	0.92	2.28	n.s.
High vs. medium and low	117	1.16	0.71	1.90	n.s.
Not stated vs. all other categories	1,036	1.21	0.94	1.56	n.s.
**Own alcohol branded merchandise**					
No or not sure	1,476	ref			
Yes	104	1.98	1.20	3.24	<0.01

Notes:
Based on never drinkers (n = 1,580) data are unweighted.
DV: Susceptibility: 1 = Susceptible (n = 830) = 0; Not susceptible (n = 750).
Test of model coefficients in final block: χ^2 (32) = 337.46, p<0.001.
Hosmer & Lemeshow for final block χ^2 (8) = 5.86, p=0.663
Nagelkerke R^2 for final block = 0.26.
Cases correctly classified in final block: 69%
[1] adjusted for all other variables in the model, Adj OR, adjusted odds ratio; ref, reference category; 95% CI, 95% confidence interval
[2] Variable not applicable to those who were classed as 'never drinkers'

For peer review only

For peer review only - http://bmjopen.bmj.com/site/about/guidelines.xhtml

STROBE 2007 (v4) Statement—Checklist of items that should be included in reports of *cross-sectional studies*

Section/Topic	Item #	Recommendation	Reported on page #
**Title and abstract**	1	(*a*) Indicate the study's design with a commonly used term in the title or the abstract	1,2
		(*b*) Provide in the abstract an informative and balanced summary of what was done and what was found	2
**Introduction**			
Background/rationale	2	Explain the scientific background and rationale for the investigation being reported	4,5,6
Objectives	3	State specific objectives, including any prespecified hypotheses	6
**Methods**			
Study design	4	Present key elements of study design early in the paper	6
Setting	5	Describe the setting, locations, and relevant dates, including periods of recruitment, exposure, follow-up, and data collection	6
Participants	6	(*a*) Give the eligibility criteria, and the sources and methods of selection of participants	6
Variables	7	Clearly define all outcomes, exposures, predictors, potential confounders, and effect modifiers. Give diagnostic criteria, if applicable	7,8,9,10
Data sources/ measurement	8*	For each variable of interest, give sources of data and details of methods of assessment (measurement). Describe comparability of assessment methods if there is more than one group	7,8,9,10
Bias	9	Describe any efforts to address potential sources of bias	7,8,9,10,11,12
Study size	10	Explain how the study size was arrived at	N/A
Quantitative variables	11	Explain how quantitative variables were handled in the analyses. If applicable, describe which groupings were chosen and why	7,8,9,10,11,12
Statistical methods	12	(*a*) Describe all statistical methods, including those used to control for confounding	11,12
		(*b*) Describe any methods used to examine subgroups and interactions	11,12
		(*c*) Explain how missing data were addressed	11,12
		(*d*) If applicable, describe analytical methods taking account of sampling strategy	11,12
		(*e*) Describe any sensitivity analyses	11,12
**Results**			

For peer review only

Participants	13*	(a) Report numbers of individuals at each stage of study—eg numbers potentially eligible, examined for eligibility, confirmed eligible, included in the study, completing follow-up, and analysed	13,14,15,17 (and all tables)
		(b) Give reasons for non-participation at each stage	13,14,15,17 (and all tables)
		(c) Consider use of a flow diagram	N/A
Descriptive data	14*	(a) Give characteristics of study participants (eg demographic, clinical, social) and information on exposures and potential confounders	12,13
		(b) Indicate number of participants with missing data for each variable of interest	13,14,15,17 (and all tables)
Outcome data	15*	Report numbers of outcome events or summary measures	13,14,15,17 (and all tables)
Main results	16	(*a*) Give unadjusted estimates and, if applicable, confounder-adjusted estimates and their precision (eg, 95% confidence interval). Make clear which confounders were adjusted for and why they were included	13,14,15,17 (and all tables)
		(*b*) Report category boundaries when continuous variables were categorized	13,14,15,17 (and all tables)
		(*c*) If relevant, consider translating estimates of relative risk into absolute risk for a meaningful time period	N/A
Other analyses	17	Report other analyses done—eg analyses of subgroups and interactions, and sensitivity analyses	13,14,15,17 (and all tables)
**Discussion**			
Key results	18	Summarise key results with reference to study objectives	17,20
Limitations	19	Discuss limitations of the study, taking into account sources of potential bias or imprecision. Discuss both direction and magnitude of any potential bias	19,20
Interpretation	20	Give a cautious overall interpretation of results considering objectives, limitations, multiplicity of analyses, results from similar studies, and other relevant evidence	17,18,19,20
Generalisability	21	Discuss the generalisability (external validity) of the study results	17,18,19,20
**Other information**			
Funding	22	Give the source of funding and the role of the funders for the present study and, if applicable, for the original study on which the present article is based	21

*Give information separately for cases and controls in case-control studies and, if applicable, for exposed and unexposed groups in cohort and cross-sectional studies.

For peer review only

Note: An Explanation and Elaboration article discusses each checklist item and gives methodological background and published examples of transparent reporting. The STROBE checklist is best used in conjunction with this article (freely available on the Web sites of PLoS Medicine at http://www.plosmedicine.org/, Annals of Internal Medicine at http://www.annals.org/, and Epidemiology at http://www.epidem.com/). Information on the STROBE Initiative is available at www.strobe-statement.org.

For peer review only

BMJ Open

Awareness of alcohol marketing, ownership of alcohol branded merchandise, and the association with alcohol consumption, higher-risk drinking, and drinking susceptibility in adolescents and young adults: A cross-sectional survey in the United Kingdom.

Journal:	*BMJ Open*
Manuscript ID	bmjopen-2018-025297.R2
Article Type:	Research
Date Submitted by the Author:	13-Dec-2018
Complete List of Authors:	Critchlow, Nathan; University of Stirling, Institute for Social Marketing<br>Mackintosh, Anne Marie; University of Stirling Institute for Social Marketing<br>Thomas, Christopher; Cancer Research UK, Cancer Policy Research Centre<br>Hooper, Lucie; Cancer Research UK, Cancer Policy Research Centre<br>Vohra, Jyotsna; Cancer Research UK, Cancer Policy Research Centre
**Primary Subject Heading**:	Public health
Secondary Subject Heading:	Addiction, Public health
Keywords:	PUBLIC HEALTH, Alcohol, Young people, Alcohol Marketing, Survey, Alcohol Advertising

SCHOLARONE™
Manuscripts

Running head: Alcohol marketing and consumption in young people in the UK

Title: Awareness of alcohol marketing, ownership of alcohol branded merchandise, and the association with alcohol consumption, higher-risk drinking, and drinking susceptibility in adolescents and young adults: A cross-sectional survey in the United Kingdom.

Running head: Alcohol marketing and consumption in young people in the UK

Authors

Nathan Critchlow[1]

Anne Marie MacKintosh[1]

Christopher Thomas[2]

Lucie Hooper[2]

Jyotsna Vohra[2]

[1] Institute for Social Marketing, Faculty of Health Sciences and Sport, University of Stirling, Stirling.

[2] Cancer Policy Research Centre (CPRC), Cancer Research UK, London.

Corresponding author

Nathan Critchlow, Institute for Social Marketing, Faculty of Health Sciences and Sport, University of Stirling, Stirling, FK9 4LA. E-mail: nathan.critchlow@stir.ac.uk; Tel: +44(0)1786467382.

Word Count: 4,478

Table and figure count: 6 (All tables)

For peer review only - http://bmjopen.bmj.com/site/about/guidelines.xhtml

Running head: Alcohol marketing and consumption in young people in the UK

Awareness of alcohol marketing, ownership of alcohol branded merchandise, and the association with alcohol consumption, higher-risk drinking, and drinking susceptibility in adolescents and young adults: A cross-sectional survey in the United Kingdom.

ABSTRACT

Objectives: To explore awareness of alcohol marketing and ownership of alcohol branded merchandise in adolescents and young adults in the United Kingdom (UK), what factors are associated with awareness and ownership, and what association awareness and ownership has with alcohol consumption, higher-risk drinking, and susceptibility.

Design: Online cross-sectional survey conducted April–May 2017.

Setting: UK.

Participants: Adolescents and young adults aged 11-19 years old in the UK (*n*=3,399).

Main outcome measures: Alcohol Use Disorders Identification Test–Consumption (AUDIT-C) (0-12) and indication of higher-risk consumption (≥5 AUDIT-C) in current drinkers. Susceptibility to drink (Yes/No) in never drinkers.

Results: Eighty-two percent of respondents were aware of at least one form of alcohol marketing in the past month and 17% owned branded merchandise. Chi-square tests found that awareness of marketing and ownership of branded merchandise varied within drinking variables. For example, higher awareness of alcohol marketing was associated with being a current drinker (χ^2=114.04, p<0.001), higher-risk drinking (χ^2=85.84, p<0.001), and perceived parental (χ^2=63.06, p<0.001) and peer approval of consumption (χ^2=73.08, p<0.001). Among current drinkers, multivariate regressions (controlling for demographics and covariates) found that marketing awareness and owning branded merchandise was positively associated with AUDIT-C score and higher-risk consumption. For example, current drinkers reporting medium marketing awareness were twice as likely to be higher-risk drinkers as those reporting low awareness (AOR=2.18, 95% CI: 1.39-3.42, p<0.001). Among never drinkers, respondents who owned branded merchandise were twice as likely to be susceptible to drinking as those who did not (AOR=1.98, 95% CI: 1.20-3.24, p=0.007).

Conclusions: Young people, above and below the legal purchasing age, are aware of a range of alcohol marketing and almost one-in-five own alcohol branded merchandise. In current drinkers, alcohol marketing awareness was associated with increased consumption and greater likelihood of higher-risk consumption. In never drinkers, ownership of branded merchandise was associated with susceptibility.

For peer review only - http://bmjopen.bmj.com/site/about/guidelines.xhtml

Running head: Alcohol marketing and consumption in young people in the UK

Keywords: Alcohol marketing; Young people; Higher-risk drinking; Alcohol Advertising; Susceptibility; Survey; Public Health; Health Policy.

Strengths and limitations of this study

- This is the first study to examine awareness of alcohol marketing and ownership of alcohol branded merchandise in a demographically representative sample of young people across the UK, including those above and below the legal purchasing age for alcohol.
- The study provides timely insight into what forms of alcohol marketing young people are aware of, how frequently they recall seeing alcohol marketing, and what factors are associated with higher awareness of alcohol marketing and ownership of alcohol branded merchandise.
- The large sample size supports robust statistical analysis to examine what relationship (if any) there is between alcohol marketing and consumption, controlling for demography and relevant covariates (e.g. peer consumption).
- The study explores the association between alcohol marketing and consumption at three levels: overall alcohol consumption and higher-risk drinking in current drinkers, and susceptibility in never-drinkers.
- The cross-sectional nature of the survey does not enable causal relationships to be drawn about the link between alcohol marketing and either consumption or susceptibility.

For peer review only

For peer review only - http://bmjopen.bmj.com/site/about/guidelines.xhtml

Running head: Alcohol marketing and consumption in young people in the UK

Awareness of alcohol marketing, ownership of alcohol branded merchandise, and the association with alcohol consumption, higher-risk drinking, and drinking susceptibility in adolescents and young adults: A cross-sectional survey in the United Kingdom.

INTRODUCTION

Adolescents and young adults (hereafter 'young people', aged 11- 19 years old) are a focal population for alcohol research because consumption at this stage of development is associated with increased drinking and risk of concomitant harms in later adulthood [1,2]. Global estimates indicate that consumption by young people is particularly high in Europe, where the proportion of current drinkers (69.5%) is higher than the five other global regions, and the proportion of lifetime abstainers is lower (15.9%) [3]. In England, it is estimated that approximately half of 11-15-year olds (44%) have consumed an alcoholic drink, one-in-ten have consumed in the past week, and nine percent have been drunk in the past month [4]. Similar estimates are reported in Scotland and Wales [5,6]. Understanding the drivers of alcohol consumption in young people is important given the immediate and long-term individual, social, and economic consequences associated with higher-risk drinking [7].

One factor routinely cited as shaping alcohol-related attitudes and behaviours in young people is marketing [8,9]. Marketing is fundamentally important to alcohol producers. It represents the primary method of communicating with new and existing consumers, can directly encourage sales, and can increase brand salience over competitors. Accordingly, alcohol companies have used highly visible marketing for over 100 years [10], with the current UK landscape characterised by a complex network of mass media marketing (e.g. television), alternative marketing (e.g. sponsorship), consumer marketing (e.g. price), and stakeholder marketing (e.g. to retailers) [9]. The importance of marketing to the alcohol industry is evidenced through their annual investment, with Diageo's global marketing expenditure approximately £1.8 billion [11]. Continued consolidation in the alcohol industry has also seen

For peer review only - http://bmjopen.bmj.com/site/about/guidelines.xhtml

the market become dominated by a small number of transnational producers, creating larger marketing budgets, economies of scale, and intense competition [12].

Content analysis research, which focuses on the marketing output as the unit of analysis, consistently reports that marketing may reach and influence young people. For example, marketing has been reported in media environments where young people may be exposed, including sports [13], social media [14], print media [15], and on-screen [16, 17]. Content research has also found that marketing may appeal to young people through creative designs, use of topical and real-world associations which may resonate with younger audiences, and by promoting positive connotations around consumption (e.g. sociability or desirable lifestyles) [18,19]. It has also been suggested that commercial marketing contains ambiguous messages about lower-risk consumption [20,21].

Systematic reviews of consumer research, which focus on the individual as the unit of analysis, provide consistent evidence that awareness of, and participation with, marketing has a causal influence on young people's consumption, including initiation and frequency of drinking [22,23]. Qualitative research has also suggested that this relationship is more complex than an 'exposure equals consumption' hypothesis, and that young people consider alcohol marketing and branding to hold rich cultural, social, and symbolic meaning [14,24,25]. Accordingly, message interpretation research has attempted to move the debate on from whether marketing is associated with consumption and onto how this influence occurs, by identifying psychological mechanisms which mediate the relationship between exposure and consumption [26,27].

In the UK, the influence of alcohol marketing on young people has been a topic of debate for decades [9,28]. These debates are further supplemented by concerns about the efficacy and effectiveness of self-regulation, the predominant approach employed to control alcohol marketing in the UK. This includes suggestions that self-regulation provides inadequate

For peer review only - http://bmjopen.bmj.com/site/about/guidelines.xhtml

restrictions, is not consistently enforced or complied with, is retrospective and slow to react to complaints, lacks meaningful sanctions, and lags behind modern marketing methods [9,28-32]. There are, however, unresolved issues which have inhibited attempts to move the debate forward. In the UK, the last large-scale assessment of young people's awareness of alcohol marketing is a decade old, was only conducted in Scotland, only sampled adolescents under the minimum purchase age, only considered overall marketing awareness (not frequency), and did not consider higher-risk consumption [33,34].

In this study, we explore frequency of awareness for alcohol marketing and ownership of alcohol branded merchandise in a demographically representative sample of young people in the UK, including those above and below the legal purchasing age. We also consider what association (if any) awareness of alcohol marketing and ownership of branded merchandise has with alcohol consumption and higher-risk drinking in current drinkers, and susceptibility to drink in never drinkers.

METHOD

Design and sample

Data come from the 2017 Youth Alcohol Policy Survey, an online cross-sectional survey conducted with 11-19-year olds in the UK (n=3,399). Responses were collected April–May 2017. The survey was hosted by YouGov, a market research company, who recruited a sample intended to be representative of the UK population from their UK panel [35]. Participants aged 16 or over were approached directly to participate, while those aged under 16 were approached through existing adult panel members known to have children. A survey weight was provided for each respondent (based on age, gender, ethnicity, region, and social grade) to enable descriptive data to be representative of the UK population. The study design was informed by

For peer review only - http://bmjopen.bmj.com/site/about/guidelines.xhtml

previous cross-sectional surveys in the UK which have explored young people's experiences of alcohol and tobacco marketing [33,36].

Measures

Demography

Alcohol consumption is not homogeneous among young people in the UK [4-7]. It is therefore important to adjust for demographic variation when examining any factors purported to be associated with consumption. In this study, age, gender, ethnicity, resident country (England, Scotland, Wales, Northern Ireland), living status, employment status, educational status, legal purchasing status for alcohol ($\geq$18 years old), and indices of deprivation (IMD), were obtained from information held about panel respondents or survey questions.

Awareness of alcohol marketing

Awareness of alcohol marketing was assessed through structured, self-reported recall, a method frequently used in consumer research [33]. Participants were prompted with the statement '*Over the last month, how often, if at all, have you seen…*' and then presented with descriptions of nine examples of alcohol marketing: (1) newspapers or magazines; (2) television; (3) billboards; (4) radio; (5) adverts on social media (e.g. YouTube, Tumblr, Facebook, SnapChat, Instagram or other social media); (6) famous people in films, music videos or TV or pictured in magazines with alcohol [celebrity endorsement]; (7) sports, games, or events sponsorship; (8) special price offers; and (9) competitions or prize draws. As per recent research [37,38], a Likert scale was used to measure frequency of noticing marketing in the past month for each of the nine examples (1=Everyday – 6=Not in the past month; Not sure).

In the UK, survey research which has measured awareness of alcohol marketing has typically used dichotomous response options for each channel (e.g. Yes/No) and used a

For peer review only - http://bmjopen.bmj.com/site/about/guidelines.xhtml

summation across these to estimate overall awareness. [33,39]. This method, however, only provides insight into breadth of marketing awareness across channels, not frequency or volume, and therefore lacks sensitivity and may underestimate awareness. To enhance accuracy in this study, the self-reported frequency of awareness for each marketing example was converted into the estimated number of days that marketing had been seen in a four-week period (i.e. 'one month'). This timeframe is consistent with previous research [40,41] and is representative of the minimum number of days in any month. For example, an answer of 'everyday' equated to 28 instances of awareness over four weeks (i.e. seven days per-week multiplied by four) and 1-2 times per-week equated to six instances over four weeks (i.e. 1.5 times per-week multiplied by four) (see Table 1 for other response options). Scores across the nine channels were summed to create an aggregate score, providing an approximation of total alcohol marketing awareness in the past month. Estimating total volume of awareness, as opposed to breadth across channels, is consistent with other recent alcohol marketing research [42,43].

In this study, an aggregate awareness score was only computed when a valid answer had been given for all nine marketing examples. To provide meaningful interpretative utility, the aggregate score for the valid sample was split into tertiles of low (aggregate score ≤16; awareness approximately every other day), medium (17-53; awareness approximately daily), and high awareness (≥54; awareness almost twice daily). If a participant answered 'not sure' to any of the nine channels they were coded as 'not stated' for the aggregate score. Indicating 'not sure' meant that a respondent's potential aggregate score was, by default, more conservative than those who provided a valid answer to all nine examples. These respondents were therefore coded as a separate 'not sure' category to avoid biasing the proportion of valid respondents considered to have low or medium awareness, or what the tertiles boundaries were.

Ownership of alcohol brand merchandise

For peer review only - http://bmjopen.bmj.com/site/about/guidelines.xhtml

Running head: Alcohol marketing and consumption in young people in the UK

Ownership of alcohol branded merchandise was measured through a single item adapted from previous research [33,44]. Participants were asked '*Do you own any merchandise (such as clothing or drinks glasses) that show an alcoholic drink brand or logo?*' (Yes/No/Not sure).

Alcohol consumption status

Participants were asked '*Have you ever had a whole alcoholic drink? Not just a sip*?' [33,34]. Those who answered 'No' were classed as never-drinkers while those who answered 'Yes' were classed as ever-drinkers.

Alcohol consumption and higher-risk drinking

Alcohol use was measured through the Alcohol Use Disorders Identification Test–Consumption (AUDIT-C), which assessed frequency of consumption, units drunk in a typical drinking occasion, and frequency of heavy episodic drinking. Responses were provided on five-point scales, with the answers for each item relative to frequency (0=Never – 4= Four or more times a week), units drunk (0=1-2 units – 4=10 units or more), or frequency of heavy episodic drinking (0=Never – 4=Daily or almost Daily). Heavy episodic drinking was classified as eight or more units in a single occasion for males, and six or more units for females (one unit=8g or 10ml of alcohol). A diagram depicting the unit content of alcoholic drinks was included to assist calculation of units. Those who answered anything other than 'never' on the first AUDIT-C item were classed as current drinkers and asked to complete the final two items. All other respondents (i.e. those stating 'never' for frequency of consumption) were classified as non-drinkers and were not asked to complete the final two items. In current drinkers, a total AUDIT-C score was computed by summing the three AUDIT-C items (0-12), with a cut-off of ≥5 used to identify higher-risk consumption [45].

For peer review only - http://bmjopen.bmj.com/site/about/guidelines.xhtml

Running head: Alcohol marketing and consumption in young people in the UK

Susceptibility

As per tobacco research, susceptibility was defined as the absence of a firm decision not to drink alcohol in the next year [36]. Never-drinkers were classified as 'non-susceptible' if they answered 'definitely no' to the question '*Do you think you will drink alcohol at any time during the next year*?' Those who answered anything other than 'Definitely no' were classified as Susceptible.

Confounding variables

Confounding factors, reported to influence consumption in young people and used in previous alcohol marketing research, were included as covariates to contextualise any association between marketing and consumption [33,34,46,47]. Frequency of consumption was measured for the mother (female carer), father (male carer), and closest friend (each scored: 1=Never – 9=Every day or almost every day; Prefer not to say; Not applicable). For all three groups, consumption was collapsed into five categories (Never, Less than monthly, Monthly or Fortnightly, At Least weekly, and Not Stated). Perceived acceptability of consumption was measured for parents and peers (each scored: 1=Total acceptable – 5=Totally unacceptable). For both groups, acceptability was converted into dichotomous categories ('Neutral or unacceptable' and 'Acceptable'). For ever drinkers, age of first drink was also measured (≤8 years old – 19 years old; Can't remember; Prefer not to say). Answers were converted into three categories (≤13 years; 14-15 years; ≥16 years).

Ethics

Ethical approval was obtained from the University of Stirling's General University Ethics Panel (GUEP59). YouGov included a lead for ethical and quality assurance, including consent,

For peer review only - http://bmjopen.bmj.com/site/about/guidelines.xhtml

post-survey debriefing and signposting to support organisations, and confidentiality and anonymity.

Patient and Public Involvement

The survey was developed following cognitive testing with a small sample (*n*=100) of young people to ensure age and cultural comprehension of the questions. Beyond this, no other patient or public involvement was undertaken.

Analysis

Data were analysed using SPSS version 23 (SPSS Inc., Chicago IL). Descriptive data were weighted so that percentages and median scores were representative of the demographic profile of the UK population. Bivariate analyses, using Chi-square tests, examined differences in level of alcohol marketing awareness and ownership of branded merchandise between the demographic and confounding variables.

A multivariate linear regression was conducted with current drinkers' AUDIT-C score as the dependent variable (0-12) and awareness of marketing and ownership of branded merchandise as the key independent variables. The following demographic and confounding variables were also included in initial blocks: age; gender; ethnicity; IMD quintile; resident country; educational status; working status; living status; frequency of mother (female carer), father (male carer) and close friend drinking; perceived parental and peer acceptability of consumption; and drink age of first drink. Categorical variables with ≥3 categories were converted into dummy (binary) variables to aid interpretation and comparison. The omitted dummy variable formed the reference category. For example, marketing awareness was a categorical variable with four levels: low, medium, high, and not stated. Four binary variables were computed: low awareness, medium awareness, high awareness, and not stated (each

For peer review only - http://bmjopen.bmj.com/site/about/guidelines.xhtml

coded Yes=1, No=0). By including medium, high and not stated in the multivariate analysis, and omitting low awareness, the reference category was low awareness. The multivariate regressions therefore indicate the association between level of consumption and medium awareness, relative to low awareness, and high awareness relative to low awareness. Reference categories for each variable are displayed in the results.

Two multivariate logistic regressions were conducted with higher-risk drinking (AUDIT-C ≥5) among current drinkers and susceptibility to drink among never-drinkers as the dependent variables. Marketing awareness and ownership of branded merchandise were the key independent variables. Where applicable, both logistic regressions controlled for the same demographic and confounding variables as the linear regression. Reference categories for categorical independent variables are indicated in the results. Where the categorical variables had three ≥3 levels, and were of an ordinal level, the SPSS contrast=difference function enabled comparison of each increasing category relative to the combined previous categories. For example, the first comparison with frequency of mother's drinking and higher-risk drinking was 'less than monthly drinking' vs. 'mother never drinks', whereas the final comparison was 'at least weekly drinking' vs. 'less often'. As the independent variables were categorical, 'not stated' responses were also included as a separate category and compared against the reference category for each variable. This enabled the maximum sample to be retained. For example, the large number of 'not stated' responses on level of marketing awareness could be compared with those for whom marketing awareness could be computed.

All multivariate analyses were conducted on unweighted data as the factors used to construct the weights were included as covariates in the models. The multivariate analyses were repeated on weighted data to check for consistency. As results for the key independent variables (marketing awareness and ownership of branded merchandise) were consistent, only the unweighted results are presented.

For peer review only - http://bmjopen.bmj.com/site/about/guidelines.xhtml

RESULTS

Sample characteristics

The weighted sample (*n*=3,399) had an average age of 15.18 years old (*SD*=2.55; range: 11-19), with three quarters (76%) below the legal purchasing age (≤18 years). There was an even distribution for gender (51% male and 49% female). The majority of the sample were White British (76%) and were evenly distributed across IMD (20% in each quintile). Most participants lived in England (84%) with the remainder from Scotland (8%), Wales (5%), and Northern Ireland (3%). Almost all participants were living at home with parent(s) or other adult family members (90%) and were in some form of education (95%).

Alcohol consumption and susceptibility

After excluding cases with missing data on drinking status (*n*=62, weighted), almost half of the weighted sample (48%; *n*=1,590) were current drinkers. Within current drinkers, the average AUDIT-C score was 4.33 (*SD*=2.77). Almost half of current drinkers (44%; *n*=707) were classified as higher-risk (≥5 AUDIT-C). After excluding cases with missing data on drinking status (*n*=62, weighted), almost half of the weighted sample (49%; *n*=1,623) were never drinkers. Within never drinkers, half were classified as susceptible (52%; *n*=841).

Awareness of alcohol marketing

The most frequent sources of marketing awareness in the past month were adverts on television (Median=6 instances per month, Inter quartile range=14), celebrity endorsement (Median=6, IQR=14), and special offers (Median=6, IQR=14) (Table 1). More than a third of respondents (range: 39-43%) had noticed marketing through these channels at least weekly. Billboard adverts (Median=2 instances per month, IQR=6), sponsorship (Median=2, IQR=6), and social

For peer review only - http://bmjopen.bmj.com/site/about/guidelines.xhtml

For peer review only

Running head: Alcohol marketing and consumption in young people in the UK

media adverts (Median=2, IQR=6) were noticed less than once a week, with at least a quarter of participants (range: 27-31%) having noticed these at least weekly. Lowest awareness was for adverts in the print press (Median=0 instances per month, IQR=6), on radio (Median=0, IQR=0), and competitions (Median=0, IQR=2). For each marketing example, a fifth or more (range: 19-29%) were not sure how often, if at all, they had come across alcohol marketing. Overall, 82% had noticed marketing through at least one channel.

[TABLE 1]

Aggregate alcohol marketing awareness

The median aggregate alcohol marketing awareness score was 32 (IQR=60), equating to noticing 32 instances of alcohol marketing in the past month (under minimum purchase age: median=28; IQR=60). When categorised into tertiles, 35% of the valid sample were classified as having low awareness (≤16 instances per month), 32% had medium awareness (17–53), and 34% had high awareness (≥54). In those under the minimum purchase age, 38% had low awareness, 31% medium, and 32% high.

Bivariate Chi-square tests found that higher awareness of alcohol marketing was significantly associated with being male, of legal purchasing age, a current drinker, a higher-risk drinker, not in education, in employment, and perceiving that parents and peers would consider it okay to consume (Table 2). Higher awareness was also associated with greater frequency of mother (female carer) consumption, $\chi^2(16)=38.25$, $p<0.001$, greater frequency of father (male carer) consumption, $\chi^2(16)=29.55$, $p<0.05$, and greater frequency of close friends drinking, $\chi^2(16)=198.51$, $p<0.001$. There was no difference in awareness by ethnicity, IMD quintile, or resident country.

For peer review only - http://bmjopen.bmj.com/site/about/guidelines.xhtml

Running head: Alcohol marketing and consumption in young people in the UK

[TABLE 2]

Owning alcohol branded merchandise

Almost a fifth of participants (17%) reported owning alcohol branded merchandise. Bivariate Chi-square tests found that ownership of branded merchandise was significantly associated with being of white British ethnicity, of legal purchase age, a current drinker, a higher-risk drinker, not in education, in employment, and perceiving that parents and peers would consider it okay to consume (Table 3). Ownership of branded merchandise was also associated with greater frequency of mother (female carer) consumption, $\chi^2(8)=44.11$, $p<0.001$, greater frequency of father (male carer) consumption, $\chi^2(8)=56.49$, $p<0.001$, and greater frequency of close friends drinking, $\chi^2(8)=178.76$, $p<0.001$. There was also an overall effect of IMD, $\chi^2(4)=15.73$, $p<0.01$, although this had no distinct pattern across escalating deprivation. There was no difference by resident country or gender.

[TABLE 3]

Association between alcohol marketing and AUDIT-C scoring

A multivariate linear regression examined the association between marketing awareness, ownership of branded merchandise, and AUDIT-C scoring in current drinkers (Table 4). After controlling for demographic and confounding factors, medium alcohol marketing awareness ($b=0.79$, 95% CI: 0.37-1.21, $p<0.001$), or high awareness ($b=0.85$, 95% CI: 0.44-1.26, $p<0.001$), compared to low awareness, was associated with higher AUDIT-C score, as was ownership of branded merchandise ($b=0.79$, 95% CI: 0.55-1.04, $p<0.001$). Of the demographic variables, being older ($p<0.001$), male ($p=0.006$), from a more affluent IMD ($p<0.01$), in education ($p<0.01$), and living independently of parents or adult family members ($p<0.001$)

For peer review only - http://bmjopen.bmj.com/site/about/guidelines.xhtml

was also associated with higher AUDIT-C score in the final model. Of the confounding variables, having a close friend who drinks at least weekly (*p*<0.001), and perceiving that parents consider it acceptable to consume (*p*<0.05) was also associated with higher AUDIT-C score. Having a first alcoholic drink at ≥16 years old (*p*<0.001) was associated with lower AUDIT-C score, compared with those who first drank aged 14–15 years.

[TABLE 4]

Association between alcohol marketing and higher-risk consumption

A multivariate logistic regression examined the association between marketing awareness, ownership of branded merchandise, and higher-risk drinking in current drinkers (Table 5). After controlling for demographic and confounding factors, medium alcohol marketing awareness (Adjusted Odds Ratio=2.18, 95% CI: 1.39-3.42, *p*<0.001), high awareness (AOR=1.43, 95% CI: 1.01-2.02, *p*<0.05), and owning branded merchandise (AOR=1.71, 95% CI: 1.31-2.22, *p*<0.001) were associated with higher-risk drinking. Of the demographic variables, being older (*p*<0.001), male (*p*<0.05), from England compared to Wales (*p*<0.05), in education (*p*<0.05), and living independently (*p*<0.05) was associated with higher-risk drinking in the final model. Of the confounding variables, increasing frequency of close friend consumption (*p*<0.001), and having had first drink aged 14-15 years old or younger (*p*<0.001), was associated with higher-risk consumption.

[TABLE 5]

Association between alcohol marketing and susceptibility to consume

For peer review only - http://bmjopen.bmj.com/site/about/guidelines.xhtml

Running head: Alcohol marketing and consumption in young people in the UK

A multivariate logistic regression examined the association between marketing awareness, ownership of branded merchandise, and susceptibility to drink in never drinkers (Table 6). After controlling for demographic and confounding variables, awareness of alcohol marketing was not associated with susceptibility, but ownership of branded merchandise was, with those who owned branded merchandise almost twice as likely to be susceptible compared to those who did not (AOR=1.98, 95% CI: 1.20-3.24, $p<0.01$). Of the demographic variables, only being white British ($p<0.01$) was associated with susceptibility in the final model. Of the confounding variables, frequency of mother (female carer) consumption ($p<0.001$), frequency of father (male carer) consumption ($p<0.05$), frequency of close friend consumption ($p<0.001$), and perceived peer approval ($p<0.001$) were associated with susceptibility.

[TABLE 6]

DISCUSSION

The findings indicate that young people in the UK are aware of a variety of alcohol marketing and almost a fifth own branded merchandise. The results also show that awareness of marketing and ownership of branded merchandise is associated with increased consumption and higher-risk drinking in current drinkers, and that ownership of branded merchandise is associated with susceptibility in never-drinkers. We address key evidence gaps in the UK by exploring frequency of marketing awareness (not just breadth of exposure) and demonstrating an association between marketing and both consumption and susceptibility in young people above and below the legal purchasing age from across the UK.

The findings are consistent with suggestions that alcohol marketing appears in contexts which may reach young people, including those under the legal purchasing age [8,9]. Awareness included mass media marketing (e.g. television), alternative marketing (e.g.

For peer review only - http://bmjopen.bmj.com/site/about/guidelines.xhtml

sponsorship and celebrity endorsement), consumer marketing (e.g. price offers), and digital media. This highlights the dynamic nature of '360-degree' marketing strategies and how they reach young people in offline and online environments [9,48]. The results extend understanding by showing how frequently young people see alcohol marketing; with at least one-in-ten reporting daily or almost daily awareness through three of the nine marketing examples. Approximately half of the sample had seen at least 32 instances of alcohol marketing per month, which equates to awareness at least once a day. Although there were expected differences in awareness between drinkers and never-drinkers [33,49], there were no differences between key demographic groups, including ethnicity, indices of deprivation, and resident country. This suggests that awareness of alcohol marketing occurs in young people across the UK, and is not isolated to a minority of demographic groups.

The results are consistent with longitudinal research which has shown a link between marketing and increased consumption in young people [22,23,34,38]. Although marketing awareness did not have an association with susceptibility in never-drinkers, ownership of branded merchandise did. Research has reported that participation with marketing has a stronger association with consumption than awareness [33,34,39,49]. Our findings therefore suggest that the effect of participation is pronounced in never-drinkers. Nevertheless, as research suggests that not all alcohol marketing or brands are equally appealing to youth [25,50], it is possible that focusing on aggregated alcohol marketing awareness (the approach in this study) may have disguised associations between individual examples of marketing and susceptibility in never drinkers. The findings also extend understanding by showing an association between marketing and consumption across young adulthood. This includes an association with susceptibility and consumption in young people under the legal purchasing age and higher-risk drinking in newly-legal drinkers. Newly legal drinkers are an important target for alcohol marketers [18] and are a key under-researched group [51]. The findings

For peer review only - http://bmjopen.bmj.com/site/about/guidelines.xhtml

Running head: Alcohol marketing and consumption in young people in the UK

therefore highlight the importance of considering the wider role that marketing plays on consumption, not just in those under the purchasing age.

Except for the Scottish Government's decision to implement minimum unit pricing [52], there has been little recent change to the UK's self-and-co-regulatory framework for alcohol marketing [53,54]. It is claimed that such self-regulatory approaches provide inadequate restrictions, are not suitably enforced, are retrospective and slow to react to complaints, and lack meaningful sanctions [9,28-32]. Although statutory regulations are cited as an alternative approach [28], studies have also questioned whether current examples, such as the Loi Évin in France, are being enforced properly or whether they reduce marketing exposure [13,37]. Further research exploring the perspectives of stakeholders involved in the production, research, consumption, and regulation of marketing would be of value to identify feasible and effective options to reduce youth exposure and form a consensus on appropriate action [55,56].

There are limitations. First, the cross-sectional design cannot identify a causal relationship between marketing and consumption, albeit a directional effect is supported by longitudinal research [22,23]. Moreover, that marketing had any association with consumption and susceptibility at all suggests that it must at least play either an initiating or reinforcing role. Second, the results are only partially representative of young adults above the legal purchasing age, albeit other research has shown similar trends in older young adults [39]. Third, the marketing channels measured are not exhaustive and, consequently, the results may underestimate awareness. Examples of omitted marketing include packaging, cinema, product placement, and a broader range of digital marketing [33,39,49]. It was also not possible to decipher whether 'not sure' responses indicated uncertainty over whether a participant had seen alcohol marketed at all through a channel or uncertainty on the frequency of awareness. This influenced the design of the regression models (to account for a 'Not sure' category). Third,

For peer review only - http://bmjopen.bmj.com/site/about/guidelines.xhtml

except for owning branded merchandise, the study only measured awareness of marketing, but not participation. As participation is reported to have a stronger effect [34,49], the results may underestimate the association between marketing and drinking outcomes. Finally, measurement of owning branded merchandise also only included two examples as prompts (clothing and drinks glasses). It is possible that different prompts may have altered recall, and that multiple items or a free text response option would have provided greater clarity on merchandise owned.

CONCLUSION

This paper makes important contributions to understanding by exploring awareness of alcohol marketing and ownership of branded merchandise by young people from across the UK, three quarters of who were under the legal purchasing age. The results highlight that '360-degree' marketing strategies have created several avenues for young people to be exposed to, or involved with, alcohol marketing, and that this is associated with consumption and higher-risk drinking in current drinkers and susceptibility in never drinkers. Further scrutiny and examination of the UK's self-regulatory approach, and viable alternatives, is needed to identify feasible, appropriate, and effective means of reducing marketing exposure in young people.

Author contribution statement: LH, CT, and JV led on study design and data acquisition. LH, CT, JV were involved in design of the study tools with support from AMM. NC and AMM planned the analysis, and this was conducted by AMM. NC and AMM led on interpretation of the results, with input from LH, CT, and JV. NC drafted the manuscript, with support from AMM on methods and results, and all authors provided feedback and approved the final version of the manuscript.

For peer review only - http://bmjopen.bmj.com/site/about/guidelines.xhtml

Running head: Alcohol marketing and consumption in young people in the UK

Acknowledgements: The authors thank YouGov for their assistance in preparing and managing delivery of the survey and data. We thank Gillian Rosenberg from the CPRC at Cancer Research UK for her role in managing the project and providing information on YouGov fieldwork. We also thank NatCen Social Research for their contribution to the original scoping studies used in the development of this project.

Funding: This work was supported by a grant from Cancer Research UK (1107098).

Declaration of interest: Nathan Critchlow is a board member of Alcohol Focus Scotland. All other authors have no conflict of interest to declare.

Data sharing statement: The raw data (deindentified participant survey responses) from the Youth Alcohol Policy is held by the Cancer Policy Research Centre at Cancer Research UK.

For peer review only

Running head: Alcohol marketing and consumption in young people in the UK

REFERENCES

1. Newbury-Birch D, Walker J, Avery L, *et al*. *Impact of alcohol consumption on young people: A systematic review of published reviews,* London: Department for Children, Schools and Family, 2009.
2. McCambridge J, McAlaney K, Rowe R. Adult consequences of late adolescent alcohol consumption: A systematic review of cohort studies. *PloS Medicine* 2011;8(2):e1000413.
3. World Health Organisation. *Global status report on alcohol and health 2014.* Geneva: World Health Organisation, 2014.
4. National Health Service Digital. *Smoking, drinking, and drug use among young people: England, 2016.* London: Health and Social Care Information Service, 2017.
5. Public Health Wales Observatory. *Alcohol and health in Wales 2014: Wales profile.* Cardiff: Public Health Wales Observatory, 2014.
6. Scottish Government. *Scottish Schools Adolescent Lifestyle and Substance Use Survey (SALSUS): Alcohol report (2015).* Edinburgh: Scottish Government, 2016.
7. Public Health England. *The public health burden of alcohol and the effectiveness and cost-effectiveness of alcohol control policies: An evidence review.* London: Public Health England, 2016.
8. Hastings G. Alcohol marketing: Grooming the next generation. *BMJ;*346:f1227.
9. Alcohol Focus Scotland. Alcohol Focus Scotland. Glasgow: Alcohol Focus Scotland, 2017.
10. Dade P. *Drink talking: 100 years of alcohol advertising,* Middlesex: Middlesex University Press, 2008.
11. Diageo. *Annual report, 2017*. London: Diageo PLC, 2017.

For peer review only - http://bmjopen.bmj.com/site/about/guidelines.xhtml

Running head: Alcohol marketing and consumption in young people in the UK

12. Collin J, Hill SE, Smith KE. Merging alcohol giants threaten global health. *BMJ* 2015;17(351):h6087.
13. Purves, RI, Critchlow N, Stead M *et al.* Alcohol marketing during the UEFA EURO 2016 football tournament: A frequency analysis. *Int J Environ Res Public Health* 2017;14(7):e704.
14. Atkinson AM, Ross-Houle KM, Begley E, *et al*. An exploration of alcohol advertising on social networking sites: An analysis of content, interactions and young people's perspectives. *Add Res Theory* 2016;25(2)91-102.
15. King C, Sigel M, Ross CS, *et al*. Alcohol advertising in magazines and underage readership: Are underage youth disproportionality exposed? *Alcohol: Clin Exp Res* 2017;41(1):1775-1782.
16. Thompson KM. Addiction media: Substances on screen. *Child Adolesc Psychiatr Clin N Am* 2005;14:473-489.
17. Lowe E, Britton J, Cranwell J. Alcohol content in the 'hyper reality' MTV show 'Geordie Shore'. *Alcohol Alcohol* 2018;53(1):337-343.
18. Hastings G, Brooks O, Stead M, *et al. "They'll drink bucket loads of the stuff": An analysis of internal alcohol industry advertising documents.* London: Alcohol Education Research Council, 2010.
19. Rhoades E, Jernigan DH. Risky messages in alcohol advertising, 2003-2007: results from content analysis. *J Adolesc Health* 2013;52(1):1116-121.
20. Jones, SC, Hall S, Kypri K. Should I drink responsibly, safely or properly? Confusing messages about reducing alcohol-related harm. *PLoS One* 2017;12(9):e0184705.
21. Smith SW, Atkin CK, Roznowski J. Are "drink responsibly" alcohol campaigns strategically ambiguous? *Health Commun.* 2006;20(1):1-11.

For peer review only - http://bmjopen.bmj.com/site/about/guidelines.xhtml

Running head: Alcohol marketing and consumption in young people in the UK

22. Anderson P, de Bruijn A, Angus K, *et al.* Impact of alcohol advertising and media exposure on adolescent alcohol use: A systematic review of longitudinal studies. *Alcohol Alcohol* 2009;44(3):229-243.
23. Jernigan DH, Noel J, Landon J, *et al.* Alcohol marketing and youth alcohol consumption: A systematic review of longitudinal studies published since 2008. *Addiction* 2018;112(S1):7-20.
24. Morey Y, Eadie D, Purves RI, *et al*. *Youth engagement with alcohol brands in the UK,* London: Cancer Research UK, 2017.
25. Purves RI, Stead M, Eadie D. "I wouldn't be friends with some if they were liking too much rubbish": A qualitative study of alcohol brands, youth identify, and social media. *Int J Environ Res Public Health* 2018;15(2):e349.
26. Austin EW, Knaus C. Predicting the potential for risky behaviour among those "too young" to drink as the result of appealing advertising. *J Health Commun.* 2000;5(1):13-27.
27. McClure AC, Stoolmiller M, Tanski SE, *et al.* Alcohol marketing receptivity, marketing-specific cognitions and underage binge drinking. *Alcohol Clin Exp Res* 2013;37(1):404-413.
28. Alcohol Health Alliance. *Health First: An evidence-based alcohol strategy for the UK.* Stirling: University of Stirling, 2013.
29. Noel JK, Babor TF, Does industry self-regulation protect young from exposure to alcohol marketing? A review of compliance and compliant studies. *Addiction* 2017;112(S1):51-56.
30. Noel JK, Babor TF, Robaina K. Industry self-regulation of alcohol marketing: A systematic review of content and exposure research. *Addiction* 2017; 112(S1):28-50.

For peer review only - http://bmjopen.bmj.com/site/about/guidelines.xhtml

Running head: Alcohol marketing and consumption in young people in the UK

31. Alcohol Concern and Alcohol Research UK. *Fit for purpose?* An analysis of the role of the Portman Group in alcohol industry self-regulation. London: Alcohol Concern and Alcohol Research UK, 2018.

32. Hastings G, Brooks O, Stead M, *et al.* (2010). Failure of self-regulation of UK alcohol advertising. *BMJ* 2010;*340:*b5650.

33. Gordon R, Harris F, MacKintosh AM, *et al.* Assessing the cumulative impact of alcohol marketing on young people's drinking: Cross-sectional data findings. *Addict Res Theory* 2011;19(1):66-75.

34. Gordon R., MacKintosh AM., Moodie C. The impact of alcohol marketing on youth drinking behaviour: A two stage cohort study. *Alcohol Alcohol* 2010;45(5):470-480.

35. YouGov. *Panel methodology*. Available from: https://yougov.co.uk/about/panel-methodology/ [accessed 6th June 2018].

36. Ford A, MacKintosh AM, Moodie C, *et al.* Cigarette pack design and adolescent smoking susceptibility: a cross-sectional survey. *BMJ Open* 2013;3:e003282.

37. Gallopel-Morvan K, Spilka S, Mutatayi C, *et al.* France's Évin law on the control of alcohol advertising: Content, effectiveness, and limitations. *Addiction* 2017;112(S1):86-93.

38. de Bruijn A, Tanghe J, de Leeuw R, *et al.* European longitudinal study on the relationship between adolescents' alcohol marketing exposure and alcohol use. *Addiction* 2016*;*111(10):17774-1783.

39. Critchlow N, Moodie C, Bauld L, *et al.* Awareness of, and participation with, digital alcohol marketing, and the association with frequency of high episodic drinking among young adults. *Drugs* 2016;23(4):328-336.

40. Synder LB, Fleming F, Slater M, *et al.* Effects of alcohol advertising exposure on drinking among youth. *Arch Pediatr Adolesc Med.* 2006;160(1):18-24.

For peer review only - http://bmjopen.bmj.com/site/about/guidelines.xhtml

Running head: Alcohol marketing and consumption in young people in the UK

41. Siegel M, Kurland RP, Castrini M, *et al.* Potential youth exposure to alcohol advertising on the internet: A study of internet version of popular television programmes. *J Subs Use* 2016;21(4): 361-367.

42. Collins RL, Martino SC, Kovalchik SA, *et al.* Alcohol advertising exposure among middle school-age youth: An assessment across all media and venues. *J Stud Alcohol Drugs* 2016;77(3):384-392.

43. Chambers T, Stanley J, Signal L, *et al.* Quantifying the nature and extent of children's real-time exposure to alcohol marketing in their everyday lives using wearable cameras: Children's exposure via a range of media in a range of key places. *Alcohol Alcohol* 2018;53(5):626-633.

44. Jones SC. Alcohol-branded merchandise ownership and drinking. *Pediatrics* 2016;137(5):1-12.

45. Research in Practice. *Scoring standardised measures – All measures and guidance.* Available from: https://www.rip.org.uk/resources/publications/practice-tools-and-guides/scoring-standardised-measures--all-measures-and-guidance-compilation-2015/ [accessed 6th June 2018].

46. Rossow I, Keating P, Felix L, McCambridge J. Does parental drinking influence children's drinking? A systematic review of prospective cohort studies. *Addiction* 2016;111(2):204-217.

47. Leung RK, Toumbourou JW, Hemphill SA. The effect of peer influence and selection processes on adolescent alcohol use: A systematic review of longitudinal studies. *Health Psychol Rev* 2014;8(4):426-457.

48. Chester J, Montgomery K, Dorfman L. *Alcohol marketing in the digital age.* Berkley, CA: Berkley Media Studies Group, 2010.

For peer review only - http://bmjopen.bmj.com/site/about/guidelines.xhtml

49. Lin E-Y, Casswell S, You RQ, Huckle T. Engagement with alcohol marketing and early brand allegiance in relation to early years of drinking. *Addict Res Theory* 2012;20(4):329-338.

50. Padon AA, Rimal, DeJong W, *et al.* Assessing youth-appealing content in alcohol advertisements: Application of a content appealing to youth (CAY) index. *Health Commun.* 2018;32(2):164-173.

51. Meier PS. Alcohol marketing research: The need for a new agenda. *Addiction* 2011;106(3):466-471.

52. Holmes J, Meier PS, Angus C, *et al.* Scotland's policy on minimum unit pricing for alcohol: The legal barriers are gone so what are the implications for implementation and evaluation? *Addiction* 2018;113(2):203-204.

53. Advertising Standards Authority. *Committee of Advertising Practitioners Code (Broadcast): 18, Alcohol,* London: Advertising Standards Authority, 2018.

54. Portman Group. *Code of practice on the naming, packaging, and promotion of alcoholic drinks* (5th Edition). London: The Portman Group, 2015.

55. Fogarty AS, Chapman S. What should be done about policy on alcohol pricing and promotions? Australians experts' views of policy priorities: A qualitative interviews study. *BMC Public Health* 2013;13:610.

56. Morey Y, Eadie D, Purves RI, *et al*. *Specialist views of alcohol marketing regulation in the UK,* London: Cancer Research UK, 2017.

For peer review only - http://bmjopen.bmj.com/site/about/guidelines.xhtml

Running head: Alcohol marketing and consumption in young people in the UK

Table 1. Awareness of alcohol marketing in the past month for young people in the UK

	Every day [28][1]	5-6 times per week [22][1]	3-4 times per week [14][1]	1-2 times per week [6][1]	Less than once a week [2][1]	Not in the last month [0][1]	Not sure	Seen at least weekly	Median Score (IQR)[2]
**Marketing channel**	%	%	%	%	%	%	%	%	
Adverts for alcohol									
… in newspapers or magazines	1.9	1.8	4.9	10.2	12.2	42.3	26.8	18.8	0 (6)
… on television	5.0	5.4	12.0	20.5	15.4	22.4	19.3	42.9	6 (14)
… on billboards	3.0	3.1	7.4	14.3	17.2	30.2	24.8	27.9	2 (6)
… on radio	1.1	1.0	2.3	5.1	7.1	54.7	28.8	9.4	0 (0)
… on YouTube, Tumblr, Facebook, Snapchat, Instagram or other social media	2.9	2.3	8.1	14.0	15.6	32.1	25.0	27.3	2 (6)
Famous people in films, music videos, on TV or pictured in magazines with alcohol	4.9	5.3	10.8	17.6	14.4	23.2	23.6	38.7	6 (14)
Sport sponsorship	2.4	3.4	7.9	17.0	17.4	27.8	24.1	30.7	2 (6)
Special offers	5.3	5.3	12.3	18.8	14.1	21.3	22.8	41.7	6 (14)
Competitions	1.4	1.2	2.8	8.2	11.9	45.6	29.0	13.6	0 (2)

Notes:
[1] Score for estimating the approximate number of days on which noticed alcohol marketing in a one month period.
[2] Median number of alcohol marketing instances noticed in a one month period.
Base: All respondents (n=3,399): weighted.

For peer review only - http://bmjopen.bmj.com/site/about/guidelines.xhtml

Running head: Alcohol marketing and consumption in young people in the UK

Table 2. Classification of alcohol marketing awareness (low, medium, and high) by demographic and confounding variables

Variable	*Valid n* (*n*= 1,411)[1]		Low awareness[2]	Medium awareness[3]	High awareness[4]	χ^2	*p*
**Gender**						9.26	<0.01
Male	735	%	32.1	30.5	37.4		
Female	676	%	37.3	32.8	29.9		
**Ethnicity**						1.09	n.s.
White British	1,082	%	34.5	32.3	33.3		
Other ethnicity	317	%	35.0	29.3	35.6		
**IMD Quintile**						10.56	n.s.
1 (most deprived)	247	%	34.4	26.3	39.3		
2	266	%	35.7	28.2	36.1		
3	288	%	36.8	31.9	31.2		
4	292	%	32.2	34.6	33.2		
5 (least deprived)	317	%	34.1	35.3	30.6		
**Country lived in**						6.89	n.s.
England	1,230	%	34.5	32.0	33.6		
Scotland	93	%	34.4	33.3	32.3		
Wales	53	%	39.6	30.2	30.2		
Northern Ireland	34	%	29.4	17.6	52.9		
**Legal purchase age**						14.10	<0.01
No	995	%	37.6	30.7	31.8		
Yes	416	%	27.4	33.7	38.9		
**Current drinker**						114.04	<0.001
No	609	%	49.9	26.9	23.2		
Yes	784	%	23.1	34.8	42.1		
**Higher risk drinker**						85.84	<0.001
No	1,027	%	41.7	29.1	29.2		
Yes	384	%	15.6	38.3	46.1		
**Education**						13.90	<0.001
Not in education	79	%	17.7	31.6	50.6		
In education	1,330	%	35.6	31.7	32.8		
**Working status**						7.93	<0.05
Not in work	1,282	%	35.6	31.6	32.8		
In work	127	%	24.4	32.3	43.3		
**Parents accept use**						63.06	<0.001
No	722	%	44.2	28.4	27.4		
Yes	689	%	24.4	35.0	40.6		
**Peer accept use**							
No	410	%	51.5	24.4	24.1	73.08	<0.001
Yes	1001	%	27.7	34.6	37.8		

Notes:
[1]Valid sample excludes those who had reported ‘not sure’ to any marketing channels); sample is weighted.
[2] Low awareness equals ≤16 instances per month (i.e. once every other day);
[3] Medium awareness equals 17-53 instances per month (i.e. almost once a day or more);
[4] High awareness equals ≥54 instances per month (i.e. almost twice a day or more);
χ^2 = Bivariate Pearson Chi Square.
Due to a large number of categories, analysis of how awareness of alcohol marketing varied by mother (female carer), father (male carer), and close friend frequency consumption only reported in text.

For peer review only

Running head: Alcohol marketing and consumption in young people in the UK

Table 3. Ownership of alcohol branded items by demographic and confounding variables

Variable	*Valid n* (n = 3,276)[1]		Own branded merchandise	χ^2	*p*
**Gender**				2.71	n.s.
Male	1,679	%	18.5		
Female	1,597	%	16.3		
**Ethnicity**				16.68	<0.001
White British	2,506	%	19.0		
Other ethnicity	745	%	12.5		
**IMD Quintile**				15.73	<0.01
1 (Most deprived)	652	%	13.5		
2	646	%	21.1		
3	644	%	17.2		
4	662	%	19.5		
5 (Least deprived)	655	%	16.0		
**Country lived in**				0.97	n.s.
England	2,759	%	17.4		
Scotland	260	%	16.2		
Wales	155	%	16.8		
Northern Ireland	103	%	20.4		
**Legal purchase age**				100.33	<0.001
No	2,488	%	13.7		
Yes	788	%	29.2		
**Current drinker**				256.07	<0.001
No	1,683	%	7.2		
Yes	1549	%	28.7		
**Higher risk drinker**				222.98	<0.001
No	2,543	%	12.3		
Yes	690	%	36.7		
**Education**				43.73	<0.001
Not in education	161	%	36.6		
In education	3,106	%	16.4		
**Working status**				31.08	<0.001
Not in work	3,028	%	16.3		
In work	239	%	30.5		
**Parents accept use**				189.06	<0.001
No	1,920	%	9.7		
Yes	1,357	%	28.2		
**Peer accept use**					
No	1,066	%	8.0	97.68	<0.001
Yes	2,210	%	21.9		

Notes:
[1]Valid sample refers to those who answered 'yes' or 'no'. Missing cases due to 'don't know' response (n = 123). Sample is weighted.
χ^2 = Bivariate Pearson Chi Square.
Due to a large number of categories, analysis of how ownership of alcohol branded merchandise varied by mother (female carer), father (male carer), and close friend frequency consumption only reported in text.

For peer review only

For peer review only - http://bmjopen.bmj.com/site/about/guidelines.xhtml

Running head: Alcohol marketing and consumption in young people in the UK

For peer review only

For peer review only - http://bmjopen.bmj.com/site/about/guidelines.xhtml

Running head: Alcohol marketing and consumption in young people in the UK

Table 4. Association between alcohol marketing awareness and AUDIT-C scoring in current drinkers

	Unstandardized coefficients				Standard coefficient		
**Variables and reference categories**	***b***	**95% CI Lower**	**95% CI Upper**	**SE**	***β***	***t***	***p***
**Constant**	-5.57	-7.05	-4.09	0.75		-7.40	<0.001
**Age**	0.43	0.35	0.51	0.04	0.30	10.70	<0.001
**Gender**							
Male (*vs. female*)	0.31	0.09	0.54	0.11	0.06	2.76	<0.01
**Ethnicity**							
White British *(vs. other)*	0.08	-0.24	0.40	0.16	0.01	0.52	n.s.
**IMD Quintile**							
(1 : most deprived to 5: most affluent)	0.11	0.03	0.20	0.04	0.06	2.68	<0.01
**Country**							
Scotland (vs. England)	-0.05	-0.40	0.31	0.18	-0.01	-0.26	n.s.
Wales & Northern Ireland (vs. England)	-0.37	-0.76	0.01	0.20	-0.04	-1.90	n.s.
**Educational status**							
In education (*vs. not*)	0.66	0.22	1.10	0.22	0.07	2.96	<0.01
**Working status**							
Working (*vs. not*)	0.31	-0.06	0.67	0.19	0.04	1.66	n.s.
**Living status**							
Living independently (*vs. with parents/adult family*)	0.87	0.54	1.20	0.17	0.12	5.17	<0.001
Not stated (*vs. with parents/adult family*)	0.42	-066	1.49	0.55	0.02	0.76	n.s.
**Frequency of mother drinking**							
Never (*vs. at least monthly*)	0.04	-0.41	0.49	0.23	0.00	0.17	n.s.
Less than monthly (*vs. at least monthly*)	-0.31	-0.63	0.00	0.16	-0.04	-1.94	n.s.
Not stated (*vs. at least monthly*)	0.42	-0.20	1.03	0.31	0.03	1.33	n.s.
**Frequency of father drinking**							
Never (*vs. at least monthly*)	0.21	-0.33	0.75	0.27	0.02	0.77	n.s.
Less than monthly (*vs. at least monthly*)	0.32	-0.08	0.72	0.20	0.03	1.57	n.s.
Not stated (*vs. at least monthly*)	0.33	-0.04	0.71	0.19	0.04	1.76	n.s.
**Frequency of close friends drinking**							
At least weekly (*vs. less often or never*)	1.44	1.19	1.69	0.13	0.26	11.32	<0.001
Not stated (*vs. less than weekly or never*)	-0.49	-0.85	-0.12	0.19	-0.06	-2.61	<0.01
**Parents' views**							
Drinking acceptable (*vs. neutral/unacceptable*)	0.29	0.01	0.57	0.14	0.05	2.06	<0.05
**Peer views**							

For peer review only

Drinking acceptable (*vs. neutral/unacceptable*)		0.08	-0.32	0.48	0.21		0.01		0.38	n.s.
**Age of first drink**										
Age 13 or under (*vs. 14 to 15 years*)		0.22	-0.07	0.51	0.15		0.04		1.50	n.s.
Age 16 or over (*vs. 14 to 15 years*)		-1.33	-1.63	-1.04	0.15		-0.21		-8.82	<0.001
Not stated (*vs. 14 to 15 years*)		-0.48	-0.89	-0.07	0.21		-0.05		-2.28	<0.05
**Alcohol Marketing Awareness**										
Medium (*vs. low awareness*)		0.79	0.37	1.21	0.21		0.11		3.70	<0.001
High (*vs. low awareness*)		0.85	0.44	1.26	0.21		0.12		4.08	<0.001
Not stated (*vs. low awareness*)		0.40	0.04	0.76	0.18		0.07		2.20	<0.05
**Own alcohol branded merchandise**										
Yes (*vs. no/not sure*)		0.79	0.55	1.04	0.13		0.13		6.30	<0.001

Notes:
Based on current drinkers: $n = 1{,}592$; data are unweighted.
DV = AUDIT-C Scoring (0-12)
Model shown is final block. Total variance explained (Adj. $R^2 = 0.36$). Durbin Watson = 2.01.
Final step model change: F (4, 1,564) = 17.44, $p<0.001$.
Overall Final model ANOVA: F (27, 1,564) = 34.33, $p<0.001$.

For peer review only

For peer review only - http://bmjopen.bmj.com/site/about/guidelines.xhtml

Running head: Alcohol marketing and consumption in young people in the UK

Table 5. Logistic regression of association between alcohol marketing and higher risk consumption among current drinkers

	Higher risk consumption among current drinkers				
	n	AOR[1]	95% CI Lower	95% CI Upper	*p*
**Age**	1,131	1.40	1.28	1.53	<0.001
**Gender**					
Female	824	Ref			
Male	768	1.32	1.04	1.68	<0.05
**Ethnicity**					
Other	228	Ref			
White British	1,364	0.97	0.69	1.37	n.s.
**IMD Quintile**					n.s.
1 (most deprived)	232	Ref			
2 vs. 1	334	1.65	1.08	2.52	<0.05
3 vs. 1,2	324	1.26	0.90	1.76	n.s.
4 vs. 1,2,3	340	1.21	0.90	1.64	n.s.
5 (most affluent) vs. 1,2,3,4	362	1.23	0.93	1.64	n.s.
**Country**					n.s.
England	1,243	Ref			
Scotland	197	0.88	0.60	1.28	n.s.
Wales	116	0.58	0.36	0.93	<0.05
Northern Ireland	36	1.35	0.60	3.01	n.s.
**Educational status**					
Not in education	146	Ref			
In education	1,446	1.61	1.01	2.55	<0.05
**Working status**					
Not working	1,374	Ref			
Working (full or part-time)	218	1.43	0.97	2.09	n.s.
**Living status**					
Living with parents/adult family	1,307	Ref			
Living independently	268	1.56	1.09	2.23	<0.05
Not stated	17	1.58	0.54	4.60	n.s.
**Frequency of mother drinking**					<0.05
Never	115	ref			
Less than monthly vs. never	284	0.47	0.27	0.79	<0.01
Monthly or fortnightly vs. less often	279	1.22	0.83	1.79	n.s.
At least weekly vs. less often	849	.93	.70	1.24	n.s.
Not stated vs. all other categories	65	1.50	.78	2.88	n.s.
**Frequency of father drinking**					n.s.
Never	76	ref			
Less than monthly vs. never	160	1.40	0.72	2.73	n.s.

For peer review only - http://bmjopen.bmj.com/site/about/guidelines.xhtml

Monthly or fortnightly vs. less often	201	0.73	0.46	1.19	n.s.
At least weekly vs. less often	964	0.83	0.61	1.15	n.s.
Not stated vs. all other categories	191	1.14	0.75	1.72	n.s.
**Frequency of close friends drinking**					<0.001
Never	72	ref			
Less than monthly vs. never	187	0.68	0.32	1.42	n.s.
Monthly or fortnightly vs. less often	463	2.20	1.44	3.35	<0.001
At least weekly vs. less often	667	3.41	2.48	4.70	<0.001
Not stated vs. all other categories	203	0.57	0.37	0.89	0.013
**Parents' views**					
Neutral or unacceptable	473	Ref			
Drinking acceptable	1,119	0.92	0.68	1.24	n.s.
**Peer views**					
Neutral or unacceptable	156	Ref			
Drinking acceptable	1,436	1.41	0.88	2.25	n.s.
**Age of first drink**					<0.001
Age 13 or under	472	Ref			
Age 14 to 15 (vs. 13 or under)	535	0.86	0.63	1.18	n.s.
Age 16 or over (vs. younger)	412	0.26	0.19	0.35	<0.001
Not stated	173	0.89	0.59	1.35	n.s.
**Alcohol Marketing Awareness**					<0.001
Low awareness	184	Ref			
Medium vs. low	274	2.18	1.39	3.42	<0.001
High vs. medium and low	326	1.43	1.01	2.02	<0.01
Not stated vs. all other categories	808	0.85	0.67	1.08	n.s.
**Own alcohol branded merchandise**					
No or not sure	1,138	Ref			
Yes	454	1.71	1.31	2.22	<0.001

Notes:
Based on current drinkers (n = 1,592); data are unweighted.
DV: Higher risk drinking on the AUDIT-C ($\geq$5), 1 = Higher risk (n = 699) and 0 = Lower risk (n = 893)
Test of model coefficients in final block: χ^2 (35) = 477.29, p<0.001.
Hosmer & Lemeshow for final block χ^2 (8) = 11.66, p = 0.17.
Nagelkerke R^2 for final block =0.35.
Cases correctly classified in final block: 72% in final block
[1] Adjusted for all other variables in the model, Adj OR, adjusted odds ratio; ref, reference category; 95% CI, 95% confidence interval

For peer review only

Running head: Alcohol marketing and consumption in young people in the UK

Table 6. Logistic regression of association between alcohol marketing and never drinkers' susceptibility to drink

	Susceptibility to drink among never drinkers				
	n	AOR[1]	95% CI Lower	95% CI Upper	*p*
**Age**	1,580	1.05	0.98	1.13	n.s.
**Gender**					
Female	791	ref			
Male	789	1.09	0.88	1.37	n.s.
**Ethnicity**					
Other	377	ref			
White British	1,203	1.51	1.12	2.03	<0.01
**IMD Quintile**					n.s.
1 (most deprived)	399	ref			
2 vs. 1	278	1.13	0.80	1.60	n.s.
3 vs. 1,2	355	1.02	0.76	1.36	n.s.
4 vs. 1,2,3	233	0.88	0.64	1.22	n.s.
5 (most affluent) vs. 1,2,3,4	315	0.84	0.63	1.11	n.s.
**Country**					n.s.
England	1,193	ref			
Scotland	191	1.14	0.80	1.61	n.s.
Wales	115	1.09	0.70	1.69	n.s.
Northern Ireland	81	0.96	0.58	1.59	n.s.
**Educational status**					
Not in education	25	ref			
In education	1,555	0.67	0.20	2.25	n.s.
**Working status**					
Not working	1,550	ref			
Working (full or part-time)	30	2.59	0.83	8.11	n.s.
**Living status**					n.s.
Living with parents/adult family	1,545	ref			
Living independently	28	0.51	0.20	1.28	n.s.
Not stated	7	1.57	0.27	9.11	n.s.
**Frequency of mother drinking**					<0.001
Never	321	ref			<0.001
Less than monthly vs. never	382	2.38	1.58	3.59	<0.001
Monthly or fortnightly vs. less often	242	1.66	1.15	2.39	<0.01
At least weekly vs. less often	560	1.47	1.11	1.94	<0.01
Not stated vs. all other categories	75	1.25	0.70	2.25	n.s.
**Frequency of father drinking**					<0.05
Never	273	ref			
Less than monthly vs. never	217	1.88	1.17	3.01	<0.01
Monthly or fortnightly vs. less often	232	1.11	0.75	1.64	n.s.

For peer review only - http://bmjopen.bmj.com/site/about/guidelines.xhtml

For peer review only

Running head: Alcohol marketing and consumption in young people in the UK

At least weekly vs. less often	686	1.39	1.05	1.84	<0.05
Not stated vs. all other categories	172	1.06	0.71	1.58	n.s.
**Frequency of close friends drinking**					<0.001
Never	922	ref			
Less than monthly vs. never	162	3.46	2.26	5.26	<0.001
Monthly or fortnightly vs. less often	80	3.32	1.66	6.65	<0.001
At least weekly vs. less often	83	0.70	0.39	1.26	n.s.
Not stated vs. all other categories	333	0.61	0.43	0.86	<0.01
**Parents' views**					
Neutral or unacceptable	1,364	ref			
Drinking acceptable	216	1.00	0.70	1.44	n.s.
**Peer views**					
Neutral or unacceptable	894	ref			
Drinking acceptable	686	2.29	1.77	2.96	<0.001
**Alcohol Marketing Awareness**					n.s.
Low awareness	279	ref			
Medium vs. low	148	1.44	0.92	2.28	n.s.
High vs. medium and low	117	1.16	0.71	1.90	n.s.
Not stated vs. all other categories	1,036	1.21	0.94	1.56	n.s.
**Own alcohol branded merchandise**					
No or not sure	1,476	ref			
Yes	104	1.98	1.20	3.24	<0.01

Notes:
Based on never drinkers (n = 1,580) data are unweighted.
DV: Susceptibility: 1 = Susceptible (n = 830) = 0; Not susceptible (n = 750).
Test of model coefficients in final block: χ^2 (32) = 337.46, p<0.001.
Hosmer & Lemeshow for final block χ^2 (8) = 5.86, p=0.663
Nagelkerke R^2 for final block = 0.26.
Cases correctly classified in final block: 69%
[1] adjusted for all other variables in the model, Adj OR, adjusted odds ratio; ref, reference category; 95% CI, 95% confidence interval
[2] Variable not applicable to those who were classed as 'never drinkers'

For peer review only

STROBE 2007 (v4) Statement—Checklist of items that should be included in reports of *cross-sectional studies*

Section/Topic	Item #	Recommendation	Reported on page #
**Title and abstract**	1	(*a*) Indicate the study's design with a commonly used term in the title or the abstract	1,2
		(*b*) Provide in the abstract an informative and balanced summary of what was done and what was found	2
**Introduction**			
Background/rationale	2	Explain the scientific background and rationale for the investigation being reported	4,5,6
Objectives	3	State specific objectives, including any prespecified hypotheses	6
**Methods**			
Study design	4	Present key elements of study design early in the paper	6
Setting	5	Describe the setting, locations, and relevant dates, including periods of recruitment, exposure, follow-up, and data collection	6
Participants	6	(*a*) Give the eligibility criteria, and the sources and methods of selection of participants	6
Variables	7	Clearly define all outcomes, exposures, predictors, potential confounders, and effect modifiers. Give diagnostic criteria, if applicable	7,8,9,10
Data sources/ measurement	8*	For each variable of interest, give sources of data and details of methods of assessment (measurement). Describe comparability of assessment methods if there is more than one group	7,8,9,10
Bias	9	Describe any efforts to address potential sources of bias	7,8,9,10,11,12
Study size	10	Explain how the study size was arrived at	N/A
Quantitative variables	11	Explain how quantitative variables were handled in the analyses. If applicable, describe which groupings were chosen and why	7,8,9,10,11,12
Statistical methods	12	(*a*) Describe all statistical methods, including those used to control for confounding	11,12
		(*b*) Describe any methods used to examine subgroups and interactions	11,12
		(*c*) Explain how missing data were addressed	11,12
		(*d*) If applicable, describe analytical methods taking account of sampling strategy	11,12
		(*e*) Describe any sensitivity analyses	11,12
**Results**			

For peer review only

Participants	13*	(a) Report numbers of individuals at each stage of study—eg numbers potentially eligible, examined for eligibility, confirmed eligible, included in the study, completing follow-up, and analysed	13,14,15,17 (and all tables)
		(b) Give reasons for non-participation at each stage	13,14,15,17 (and all tables)
		(c) Consider use of a flow diagram	N/A
Descriptive data	14*	(a) Give characteristics of study participants (eg demographic, clinical, social) and information on exposures and potential confounders	12,13
		(b) Indicate number of participants with missing data for each variable of interest	13,14,15,17 (and all tables)
Outcome data	15*	Report numbers of outcome events or summary measures	13,14,15,17 (and all tables)
Main results	16	(*a*) Give unadjusted estimates and, if applicable, confounder-adjusted estimates and their precision (eg, 95% confidence interval). Make clear which confounders were adjusted for and why they were included	13,14,15,17 (and all tables)
		(*b*) Report category boundaries when continuous variables were categorized	13,14,15,17 (and all tables)
		(*c*) If relevant, consider translating estimates of relative risk into absolute risk for a meaningful time period	N/A
Other analyses	17	Report other analyses done—eg analyses of subgroups and interactions, and sensitivity analyses	13,14,15,17 (and all tables)
**Discussion**			
Key results	18	Summarise key results with reference to study objectives	17,20
Limitations	19	Discuss limitations of the study, taking into account sources of potential bias or imprecision. Discuss both direction and magnitude of any potential bias	19,20
Interpretation	20	Give a cautious overall interpretation of results considering objectives, limitations, multiplicity of analyses, results from similar studies, and other relevant evidence	17,18,19,20
Generalisability	21	Discuss the generalisability (external validity) of the study results	17,18,19,20
**Other information**			
Funding	22	Give the source of funding and the role of the funders for the present study and, if applicable, for the original study on which the present article is based	21

*Give information separately for cases and controls in case-control studies and, if applicable, for exposed and unexposed groups in cohort and cross-sectional studies.

For peer review only

Note: An Explanation and Elaboration article discusses each checklist item and gives methodological background and published examples of transparent reporting. The STROBE checklist is best used in conjunction with this article (freely available on the Web sites of PLoS Medicine at http://www.plosmedicine.org/, Annals of Internal Medicine at http://www.annals.org/, and Epidemiology at http://www.epidem.com/). Information on the STROBE Initiative is available at www.strobe-statement.org.

For peer review only
